# A compositional semantics for Venn diagrams

**DOI:** 10.1007/s10988-026-09461-3

**Published:** 2026-06-05

**Authors:** Bryan Pickel, Brian Rabern

**Affiliations:** 1 University of Glasgow, Glasgow, Scotland; 2 University of Edinburgh, Edinburgh, Scotland

**Keywords:** Diagrams, Venn diagrams, Visual representation, Compositional semantics

## Abstract

This paper examines Venn diagrams as a case study of visual representation. Venn diagrams have a clear formal structure and clearly defined correctness conditions. The question for this paper is whether these diagrams have a recursive syntax and corresponding compositional semantics by analogy with those developed for formal or natural languages. Existing research pointedly fails to offer a compositional semantics for Venn diagrams. This might suggest a fundamental cleavage between linguistic representation and the kind of visual representation exhibited by diagrams. Or, it might indicate a case of representation that recalcitrantly resists compositional analysis. This paper presents two approaches to the syntax of Venn diagrams: a region-first approach and a cell-first approach. It then develops a compositional semantics for each approach. The paper therefore helps to locate the similarities and differences more precisely between linguistic representation and the kind of representation exhibited by Venn diagrams. The two approaches to the syntax and semantics for Venn diagrams also suggest new avenues for interpreting other forms of visual representation.

## Introduction

Compositionality is widely regarded as a central guiding principle in semantic theory. In particular, the “rule-to-rule” conception has deep roots in the philosophy of language and remains a standard assumption in the dominant tradition of linguistics (see Montague, 1973; Partee, 1984). Rule-to-rule compositionality begins with the thought that complex expressions are derived from the more basic expressions by applying a sequence of syntactic formation rules. Each syntactic rule for deriving a complex expression corresponds to a rule for specifying the meaning of the derived expression in terms of the meanings of the more basic expressions.

The principle of compositionality is a high-level methodological hypothesis in linguistics (see Dever, 1999; Partee, 1984). It places constraints on the shape of semantic theories, and time and again, data that initially appear resistant to compositional treatment have been successfully analyzed within this framework. Such analyses frequently yield novel predictions.

In addition to its role as a methodological hypothesis, the principle is also motivated by the fact that communication in language is productive and systematic. Language users can produce and understand indefinitely many sentences on the basis of their understanding of a limited number of expressions. This ability is explained if the language user has mastered the rules for specifying the derived sentences in terms of the basic expressions and the corresponding rules for deriving the meanings of the derived sentences in terms of the meanings of the basic expressions. Thus, theories are often rejected or modified on account of failing to deliver a compositional analysis of the target phenomenon.

Beyond linguistic representation, information can also be shared by other forms of media, including visual media such as diagrams, maps, and pictures. However, it is regularly taken for granted that visual representations lack the kind of “syntactic structure” appropriate to the notion of compositionality operative in semantics (Fodor, 2007 and Greenberg, 2011). We will approach this issue by exploring the relatively easy case of Venn diagrams.

The groundbreaking work on Venn diagrams by Sun-Joo Shin (1994, 2011; cf. Hammer & Danner, 1996) has been taken as a model of a rigorous semantics for visual representations that is based on a radically different underlying structure from natural language. For example, Camp appeals to Shin’s semantics for Venn diagrams in order to propose that there are productive and systematic representations that do not exhibit the structure of a language.^1^

Diagrammatic representational systems, such as Venn diagrams, are formed by combining formal elements like circles, dots, and lines according to systematic rules which determine the representational content of the whole. Further, they are governed by formal rules of inference which are sound and complete, up to expressive equivalence with monadic first-order predicate logic (Shin, 1994). But the elements and combinatorial rules for diagrams are very different than those for sentences. (Camp, 2007: 153-4)

Camp is correct that the syntactic/semantic theory of Shin (1994) (and following her Hammer & Danner, 1996) differs from the common syntactic/semantic theories of linguistic representation. In particular, Shin’s account is not compositional. Shin’s account specifies each Venn diagram as the output of a syntactic rule applied to simpler representations. However, Shin’s semantics does not specify the meaning of a diagram in terms of the meanings of the simpler representations from which it derives. Rather, Shin’s semantic theory appeals to the intrinsic structure of a Venn diagram in order to specify its semantic value. This approach is satisfactory for Shin’s purposes of defining a notion of logical consequence. However, the failure of compositionality suggests that Venn diagrams, and perhaps visual representations more generally, exhibit a kind of representation that is fundamentally different from linguistic representation.

Do they? Or can the principle of compositionality operative in linguistics be applied to forms of visual representation, and Venn diagrams in particular? We will provide a theory whereby the following hold:

(i) Each Venn diagram can be specified by a finite number of applications of a finite number of syntactic rules to an enumerable vocabulary.
(ii) The syntactic derivation of a Venn diagram can be specified in a parse tree on analogy with the syntactic derivation of a formula of a natural or formal language.
(iii) The truth conditions of a Venn diagram can be compositionally specified in terms of its syntactic derivation. The semantics is compositional in that each syntactic rule in the derivation of a diagram corresponds to a semantic rule in the derivation of its truth conditions.

To put our point in a bold Montagovian voice: “There is in our opinion no important theoretical difference between *linguistic* representation and the kind of *diagrammatic* representation exhibited by Venn diagrams; indeed we consider it possible to comprehend the syntax and semantics of both kinds of representation with a single natural and mathematically precise theory” (Montague, 1970: 373, adapted to our discussion).

In a softer voice, the standard semantic theories for Venn diagrams look very different from standard semantic theories for natural or formal languages, in part because the former are not compositional. Offering a compositional semantics for Venn diagrams may help enable us to see in what respects the kind of diagrammatic representation exhibited by Venn diagrams is similar to linguistic representation and in what respects it is different.^2^

In addition to teaching a lesson about the nature of diagrams, this project may also teach us about the nature of compositionality itself. It will be shown that representations in a very different format from languages can yield a very similar compositional analysis. This will help separate inessential aspects of a compositional semantics—such as unique readability—from essential presuppositions.

## Venn diagrams

In his 1880 article “On the Diagrammatic and Mechanical Representation of Propositions and Reasonings”, John Venn introduced a scheme of diagrammatic representation which he took to be an improvement over traditional Eulerian circles—the basic system of representation was further developed in Venn (1894). Paradigmatically, a Venn diagram is given by a configuration of overlapping basic regions. The basic regions represent sets in a domain. The intersection, union, or relative complement of regions in a diagram represent the intersection, union, or relative complement of the sets in the domain represented by these regions. For example, consider a diagram with three basic regions $A_1$, $A_2$, and $A_3$, whose regions labeled *a* through *h* comprise the 8 compartments or cells of the diagram (see Diagram 1). The regions of a Venn diagram may then be decorated to indicate that the region of the diagram represents either an empty or a non-empty subset of the domain.

**Diagram 2:**
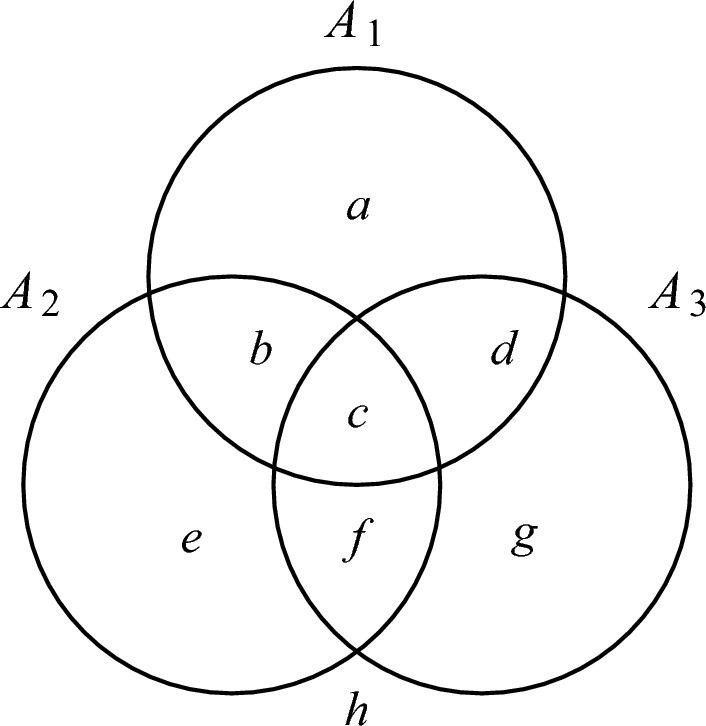
Primary Venn diagram with labeled cells

### Diagrams and destruction

To indicate that the subset of the domain represented by a region is empty—or, as Venn (1880) says to indicate “destruction”, one “shades” the region. Thus, Diagram 2 represents a domain with three subsets—represented by the regions $A_1$, $A_2$, and $A_3$. The fact that the intersection of the regions ($\bigcap \{A_1,A_2,A_3\}$) is shaded represents that the intersection of the sets represented by $A_1$, $A_2$, and $A_3$ is empty.

**Diagram 3:**
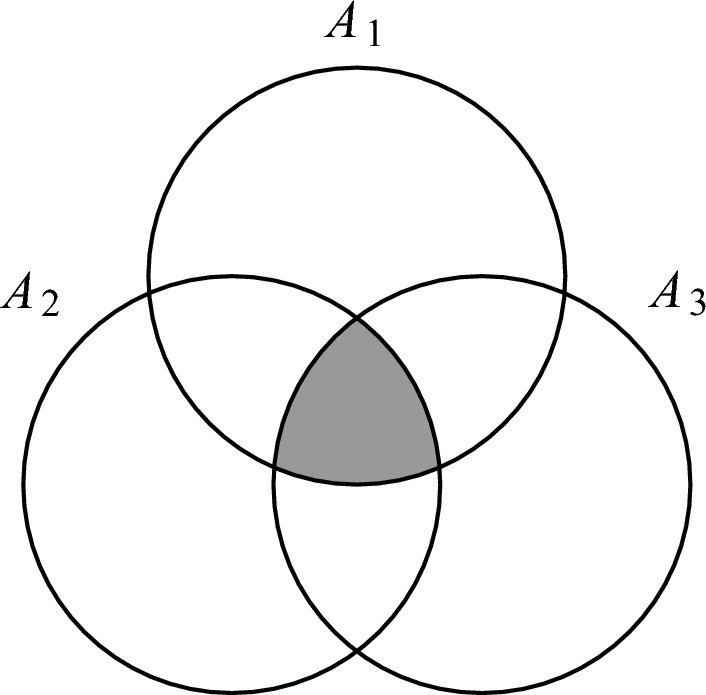
A Venn diagram with shaded region

The emptiness of a region distributes over its subregions. So in Diagram 3, the entire intersection of the regions $A_1$ and $A_2$ is shaded. This represents that the intersection of the sets represented by regions $A_1$ and $A_2$ is empty. As a result, both subregions $\bigcap \{A_1,A_2,A_3\}$ and $\bigcap \{A_1,A_2\} -A_3$ are shaded, indicating that these regions represent the empty set. Thus, Diagram 3 entails Diagram 2.

**Diagram 4:**
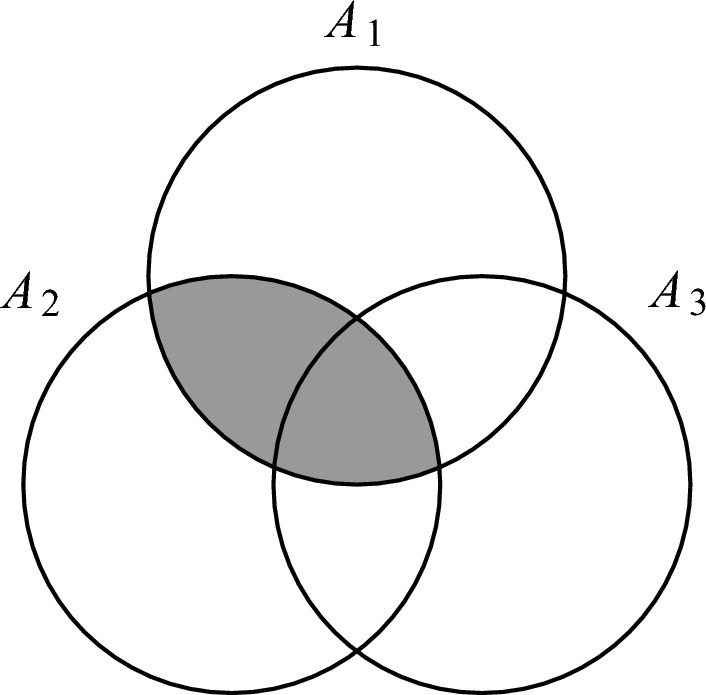
Destruction distributes

Diagram 3 can be seen as constructed in two different ways: one is *cell-first*, the other is *region-first*. The region-first construction of Diagram 3 begins with an undecorated diagram—what is called a *primary* diagram—with basic regions $A_1$, $A_2$, and $A_3$ and then “all-at-once” marks the entire region that is the intersection of $A_1$ and $A_2$. The cell-first construction, by contrast, begins with the primary diagram and then marks the intersection in a “piecemeal” manner. First, the cell defined by $\bigcap \{A_1, A_2,A_3\}$ is marked to yield Diagram 2. Then the cell $\bigcap \{A_1,A_2\} -A_3$ is marked to yield Diagram 3. The result is the same, but the two constructions correspond to two ways of developing the syntax and compositional semantics for “destruction” in Venn diagrams.

### Diagrams and salvation

A shaded region represents emptiness but a non-shaded region does not represent non-emptiness. Thus, Venn diagrams were initially not equipped to represent that a certain region was non-empty. As Venn noted the representation of non-emptiness requires “some additional form of diagrammatical notation” (Venn 1883: 599).^3^ Venn initially suggested different kinds of marking or shading: one kind for “destruction” and one kind for “salvation”. That is, Venn suggested that one could represent that the intersection of the sets represented by $A_1$ and $A_2$ was non-empty by shading them in a new way such as in Diagram 4.

**Diagram 5:**
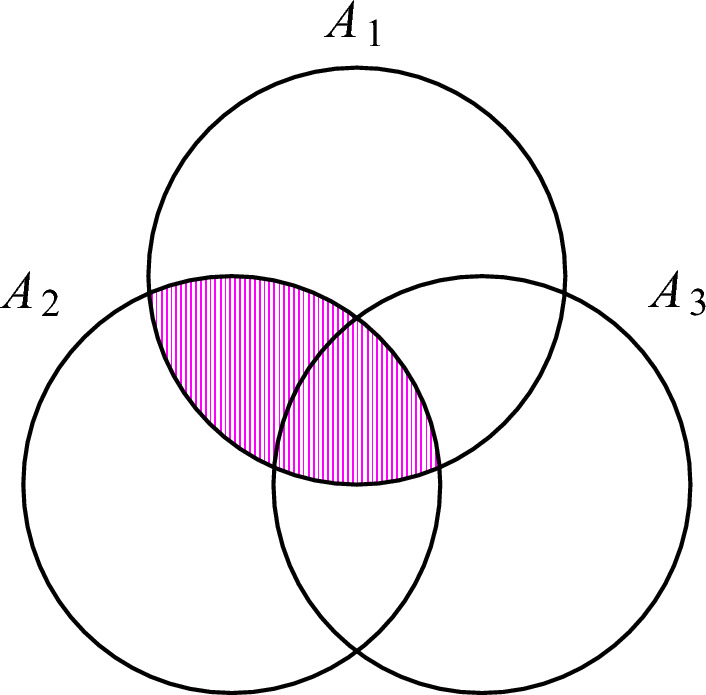
Salvation marking

In line with Venn’s convention for destructive shading, the most natural way of interpreting Venn’s suggestion for salvation marking is as a region-first construction. Diagram 4 is derived as follows: begin with a primary diagram and then existentially mark the entire intersection of $A_1$ and $A_2$.

In fact, there is a difficulty with viewing Diagram 4 as also having a cell-first construction. In particular, we must distinguish two cases: the case in which each of the constituent cells $\bigcap \{A_1,A_2,A_3\}$ and $\bigcap \{A_1,A_2\} -A_3$ represent non-empty sets and the case in which their union (namely, $\bigcap \{A_1,A_2\}$) represents a non-empty set. This difficulty doesn’t arise in the case of destructive shading. Diagram 3 represents that *nothing* corresponds to the intersection of $A_1$ and $A_2$. Notice that this is equivalent to saying that *nothing* is represented by *either* of the constituent cells of the intersection (namely, $\bigcap \{A_1,A_2,A_3\}$ and $\bigcap \{A_1,A_2\} -A_3$). Commenting on this difference between destruction and salvation, Venn says “destruction is distributive” whereas “salvation is only alternative” (Venn, 1880: 600).

In order to indicate that the constituent cells $\bigcap \{A_1,A_2,A_3\}$ and $\bigcap \{A_1,A_2\} -A_3$ each represent non-empty sets, we will need a different color (or hatching) for each region, as in the Diagram 5 constructed by first marking cell $\bigcap \{A_1,A_2,A_3\}$ and then marking cell $\bigcap \{A_1,A_2\} -A_3$ with a *new* color.^4^

**Diagram 6:**
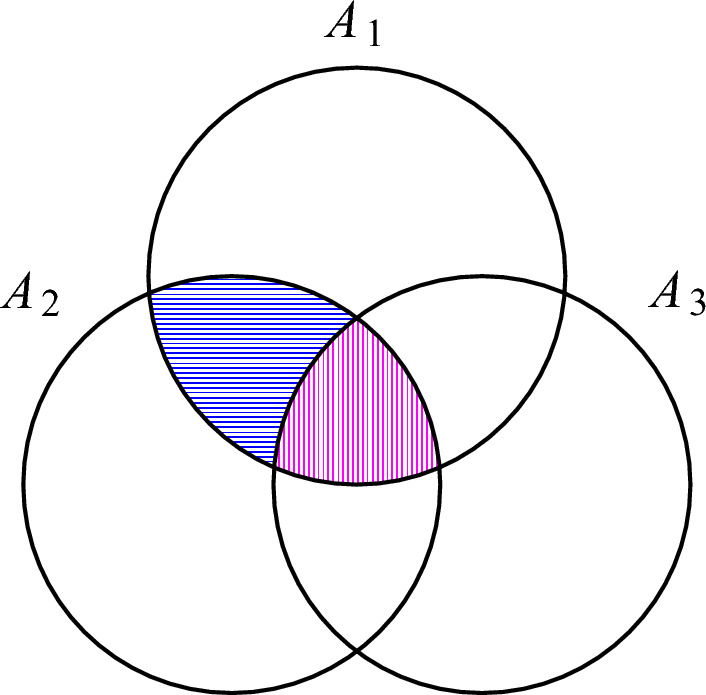
Multiple salvation

Diagram 5 represents a conjunction of existential claims. By contrast, Diagram 4 represents only that at least one of the sets represented by $\bigcap \{A_1,A_2,A_3\}$ and $\bigcap \{A_1,A_2\} -A_3$ are non-empty.

Venn next considers an alternative proposal that is most naturally construed as a *cell-first* construction. Venn says, “perhaps the simplest way is to use numerals to mark the compartments one or other of which is to be secured by each particular proposition” (Venn, 1894: 132). On this new proposal, one places occurrences of a numeral in various “minimal” regions or *cells* of a Venn diagram. The diagram then represents that the set associated with the union of these cells is non-empty. For example, Diagram 6 places the numeral ‘1’ in both the region $\bigcap \{A_1,A_2,A_3\}$ and the region $\bigcap \{A_1,A_2\} -A_3$.

**Diagram 7:**
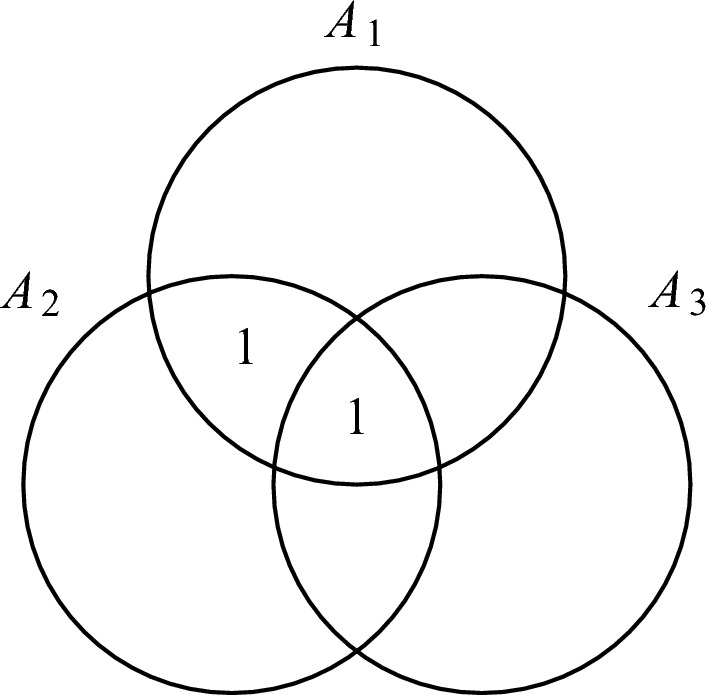
Venn’s variables

This represents that the union of all the cells containing ‘1’ correspond to a non-empty subset of the domain. That is, something is either in the intersection of $A_1$ and $A_2$ (but not in $A_3$) or something is in the intersection of all of $A_1$, $A_2$, and $A_3$. A single Venn diagram may have multiple “chains” of numerals. Thus, Diagram 7 contains a chain associated with the numeral ‘1’ and the numeral ‘2’.

**Diagram 8:**
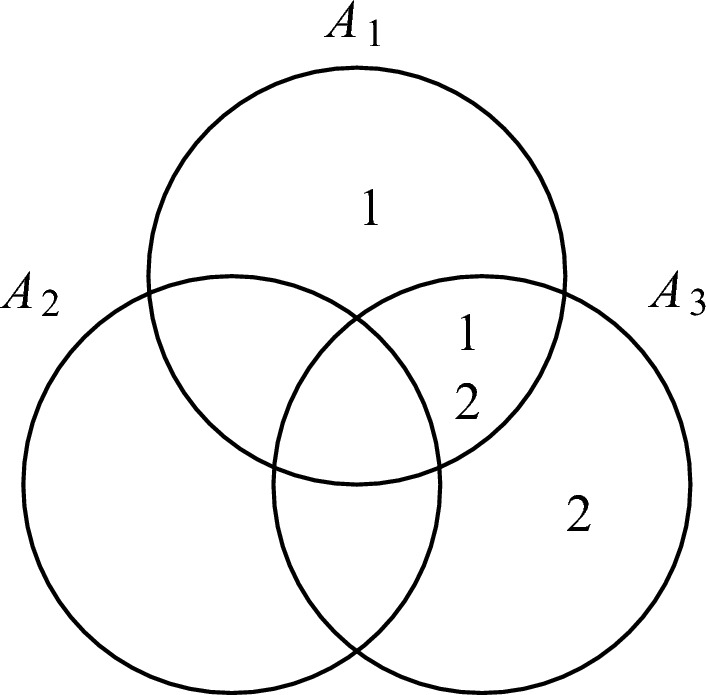
Chains of variables

Each region containing a chain of numerals indicates that the set the region represents is non-empty. The convention for using numeral chains for “salvation” is combined with the initial convention of shading for “destruction” to arrive at the complete system of representation (as in 8) that we will call a Venn diagram.^5^

**Diagram 9:**
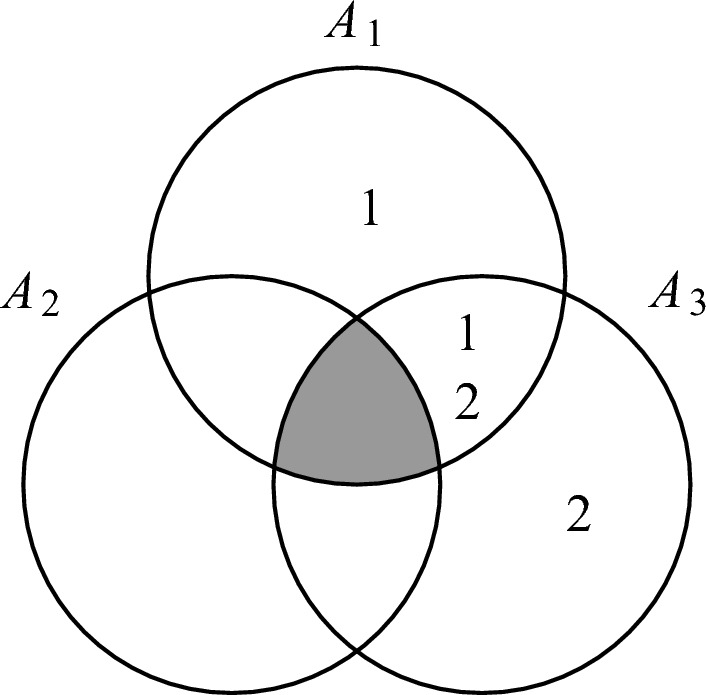
A Venn diagram

Later, Peirce proposed an alternative notation that constructs chains by placing *x*’s in cells and connecting them by lines. The region containing a maximal chain represents a non-empty set. Thus, Peirce’s Diagram 9 is equivalent to Venn’s Diagram 8.^6^

**Diagram 10:**
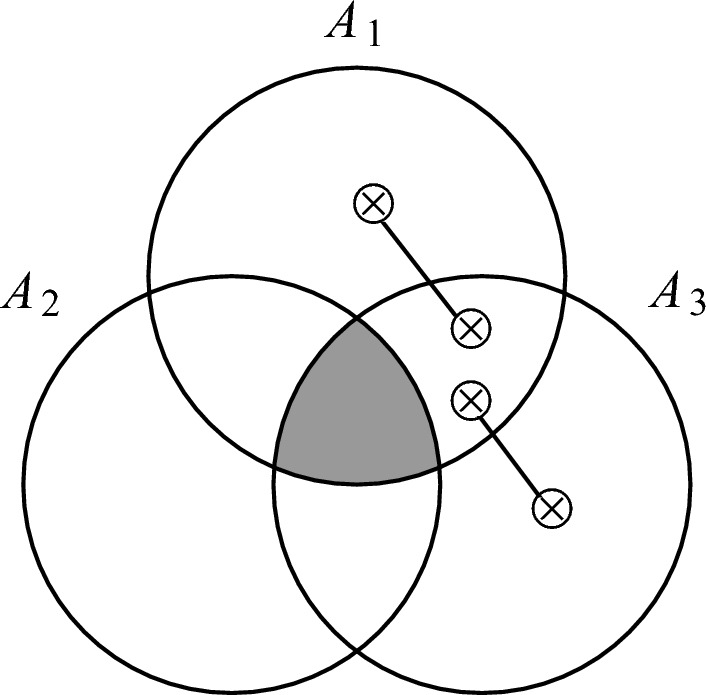
Peircian chains

In her pioneering work, Shin follows Peirce’s convention regarding the encoding of existential information in Venn diagrams. We will first follow Venn by making use of variables, i.e. numbers, to indicate non-emptiness. We will then show how our approach can be extended to capture Peirce’s notation by applying a transformation rule to replace occurrences of a single variable by a Peircian chain.

## Diagrams and compositionality

Compositionality in general is the thesis that the meaning of a complex expression is determined by the meanings of its syntactic constituents and their arrangement. This thesis has many different implementations in linguistics, logic, cognitive science, and philosophy (e.g., Pickel & Szabó, 2025).^7^ In this piece we focus on a dominant interpretation in formal semantics, rule-to-rule compositionality.^8^ Rule-to-rule compositionality assumes that every complex expression is derived by applying a syntactic formation rule to simpler expressions. Each such formation rule is paired with a corresponding semantic evaluation rule.

**Rule-to-rule compositionality**. *For each syntactic formation rule* *R*, *there is a semantic evaluation rule* $S_R$, *and if a complex representation* α *is derived by applying rule* *R* *to simpler representations* $\beta _1,\ldots ,\beta _n$, *then the meaning of* α *is the result of applying* $S_R$ *to the meanings of* $\beta _1,\ldots ,\beta _n$.

This is the standard interpretation of the compositionality thesis; it has been called “Frege’s Principle” and, more recently, Pagin and Westerståhl (2010a) simply call it *basic compositionality*.^9^

The history of semantics is littered with examples of purportedly intractable exceptions to compositionality that eventually yielded to compositional analysis (cf. Portner & Partee, 2008). One salient example is Kamp’s (1982) discourse representation theory which was meant to explain anaphoric connections that extended beyond the sentence boundary such as the anaphoric pronouns ‘he’ and ‘it’ in the following sequence of sentences:

**Figure:**
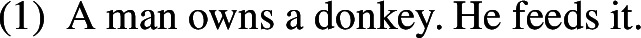

Kamp’s original theory was non-compositional. This set off a race to develop a compositional version of the theory issuing in works such as Zeevat (1989), Asher (1993), and Muskens (1996). Compositional implementations of non-compositional semantics have often led to further predictions and models in semantic theory, which indirectly confirm (rule-to-rule) compositionality as a working hypothesis.

In contemporary formal semantics, compositionality functions precisely in this way: it provides the framework within which semantic theories are developed and new data are interpreted. Within this framework, theories become empirically testable. Compositionality itself, however, is not directly testable, but only in conjunction with additional assumptions about syntax, semantics, and pragmatics (cf. Partee, 1984, 14).

### The role of compositionality

This methodological role becomes clearer once we examine how, on the rule-to-rule conception, compositionality links syntactic structure to semantic interpretation. On this conception, the meaning of each complex expression is determined by the meanings of its immediate constituents—the configuration of expressions in the derived output cannot make a difference.^10^ Let us illustrate this using the example sentence (2).

**Figure:**
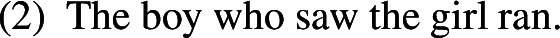

Compositionality presupposes that this sentence has a syntactic derivation. Most syntactic derivations will say that this sentence is formed by combining a noun (or determiner) phrase (NP) ‘the boy who saw the girl’ with the verb phrase (VP) ‘ran’. This entails that the meaning of (2) must be a function of the meanings of the NP ‘the boy who saw the girl’ and the VP ‘ran’ and of the rule that allows one to combine an NP and VP.^11^

Notice that the string displayed in (3) appears as a part of the output string displayed in (2).

**Figure:**
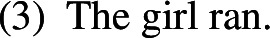

However, the sentence (3) does not appear in the *syntactic derivation* of sentence (2). This means that (3) is not a syntactic constituent of the sentence (2). Compositionality requires that the meaning of (2) be specified only in terms of the meanings of its constituents and construction rules. So, the meaning of ‘the boy who saw the girl ran’ can’t depend on the meaning of ‘the girl ran’ even though the latter is an orthographic part of the resulting sentence. Notice how strange it would be to say that the meaning of ‘the boy who saw the girl ran’ depends on the meaning of ‘the girl ran’ even though the latter is not a syntactic constituent.^12^

We emphasize this example because, as we shall argue, existing semantic accounts of Venn diagrams fall short of genuine compositionality. As discussed in §7, these approaches derive semantic interpretation directly from the *topographic* structure of the final diagram rather than from the syntactic derivation that generates it. This risks allowing non-constituents to play a role in determining meaning—much as a linguistic theory would if it allowed the orthographic fragment ‘the girl ran’ to contribute to the meaning of ‘the boy who saw the girl ran’.

The notion of rule-to-rule compositionality can be applied to representations other than sentences. What is required is that these representations are “built-up” or derived by the sequential application of a set of syntactic rules starting with some basic representations (Hodges, 1998). The standard semantics of Shin (1994), Hammer and Danner (1996), and others does satisfy this syntactic presupposition. On these approaches, every diagram is specified by a sequence of operations performed on the basic regions. We will provide our own method for constructing each Venn diagram from the basic regions, distinguishing between two different approaches that are blurred in the standard approach.

But fundamentally, our difference from these prior approaches is *semantic* and not syntactic. We agree that the Venn diagram can be specified as a function of operations applied to its basic regions. We also agree that basic regions should be taken to represent subsets of the domain. We diverge on whether the content of the Venn diagram is to be specified as the result of a sequence of operations on the contents of the basic regions. We will offer a semantic theory for Venn diagrams that is compositional. In doing so, we hope to distinguish aspects of Venn diagrams that might be run together in approaches that do not aim at compositionality.

In addition to its role as a high-level methodological principle in semantics, compositionality is sometimes also invoked to explain humans’ ability to understand novel expressions within a representational system (productivity) and to form new representations by recombining familiar ones (systematicity). Human competence with Venn diagrams is likewise productive and systematic: users can interpret novel diagrams after exposure to only a few examples, and can generate new diagrams by recombining familiar elements. This raises the possibility that competence with Venn diagrams might also be explained by presupposing that humans process such diagrams in a way analogous to a compositional semantics.

To develop this hypothesis, however, we must first establish that a compositional semantics for Venn diagrams is even available. If a representational system resists compositional analysis, there is no reason to suppose that the human cognitive system implicitly encodes one. Moreover, to investigate whether any particular semantics could be psychologically realized, we must have the relevant semantic details explicitly articulated. The same representational set may correspond to many different syntactic theories, and different semantic theories will yield different predictions about the meanings of constituent expressions. Thus, if we presuppose that some compositional semantics is encoded by the cognitive system, distinct syntactic and semantic theories will lead to distinct empirical predictions.

We have sketched two ways of construing the construction of Venn diagrams: *region-first* and *cell-first*. We will develop a syntactic theory for Venn diagrams corresponding to each approach and offer a compositional semantics for each. Existing syntactic theories in the literature tend to “mix and match” these systems. For example, Shin (1994, 57, rules 3–5) offers a region-first syntax for *destruction* and a cell-first syntax for *salvation*, whereas Hammer & Danner (1996, 466, rules 3–4) offers the reverse. One further aim of this paper is to show that these two approaches to the syntax of Venn diagrams can be cleanly separated. On the assumption that cognitive systems encode a recursive syntax and semantics for Venn diagrams, the two resulting theories might be expected to yield different testable predictions. But we make no claims here about the cognitive reality of the proposed systems—we are concerned instead with the prior theoretical questions.

## The region-first approach

**Diagram 11:**
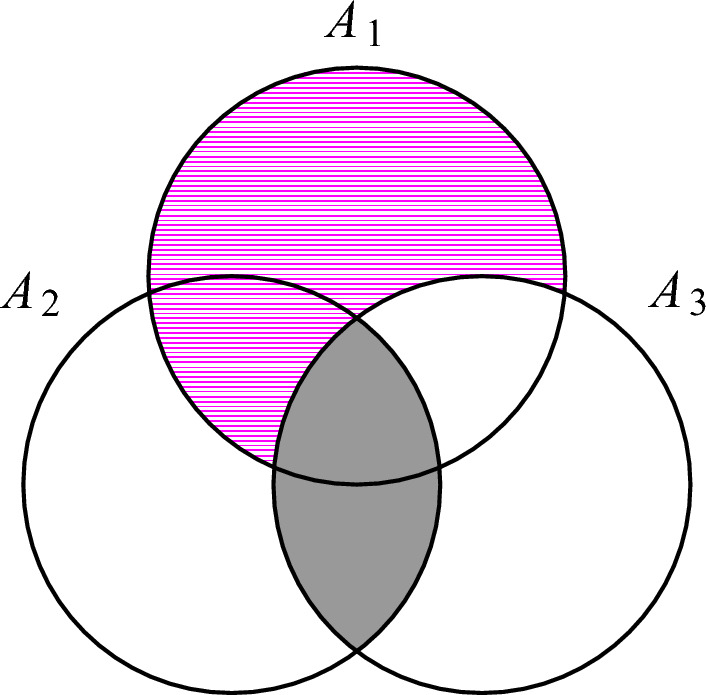
$$\bigg \langle \big \{A_1,A_2,A_3\big \}, A_2\,{\cap }\,A_3,\,\big \{A_1\,{-}\,A_3\big \}\bigg \rangle $$

A Venn diagram is a certain formal object. Each diagram consists of a set of basic regions in a background region. Out of these basic regions sub-regions can be defined by set-theoretic intersection, union, and relative complement. On the region-first approach, these sub-regions are then decorated by a single destruction marker and various salvation markers. Formally then, a Venn diagram is identified by a triple $\langle B, \Delta , \Sigma \rangle$, where *B* is the set of its basic regions, Δ is its destroyed region, and Σ is a set of its saved regions. Thus, the Diagrams 10 and 11 can be specified by the corresponding triples.

**Diagram 12:**
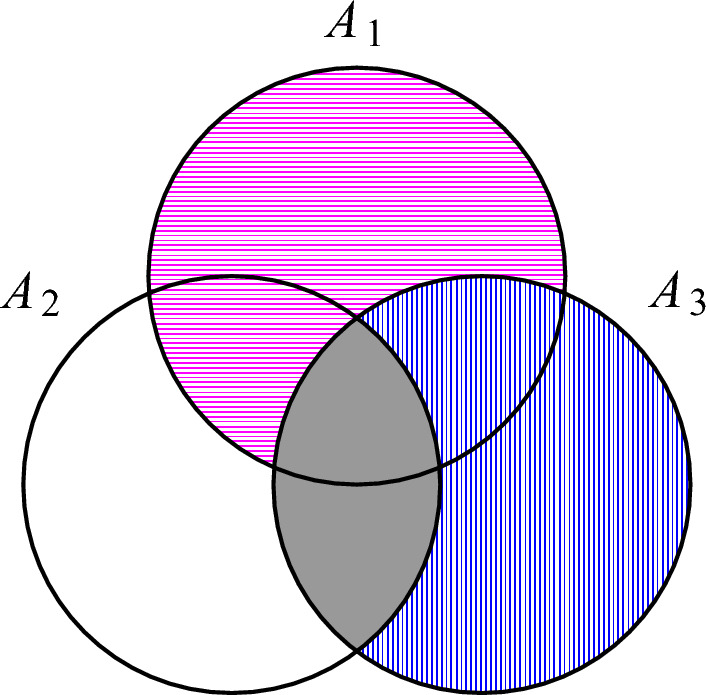
$$\bigg \langle \big \{A_1,A_2,A_3\big \}, A_2\,{\cap }\,A_3,\,\big \{A_1\,{-}\,A_3, A_3\,{-}\,A_2\big \}\bigg \rangle $$

For diagram $D = \langle B, \Delta , \Sigma \rangle$, we will write $D_B$, $D_\Delta$, and $D_\Sigma$ to indicate the relevant element of the triple.

### The region-first syntax

The syntax for the region-first approach is fairly straightforward. The basic vocabulary consists of a set of basic regions $X_1, X_2,\ldots$ and a background *R*. We will say that there is an operation $\texttt {form}$ that derives a primary diagram from a (potentially empty) sequence $X_1,\ldots , X_n$ of basic regions provided these regions satisfy what is known as the *mutual overlap constraint*. The way Venn put the intuitive constraint was as follows:

“What we ultimately have to do is to break up the entire field before us into a definite number of classes or compartments which are mutually exclusive and collectively exhaustive.” (Venn 1894, 111)

The constraint simply guarantees that the cells of the diagram are non-empty.^13^

#### Definition 1

A (non-empty) set of basic regions $\{X_1,\ldots , X_n\}$ of the plane *R* meets the mutual overlap constraint iff for all Γ: if $\Gamma \subseteq \{X_1,\ldots , X_n\}$, then $\bigcap \big (\Gamma \cup \{R\}\big ) - \bigcup \big (\{X_1,\ldots , X_n\}- \Gamma \big ) \ne \emptyset$.

Formally, $\texttt {form}$ is a function that takes some basic regions $X_1,\ldots X_n$ to a triple $\big \langle \{X_1,\ldots X_n\}, \emptyset ,\emptyset \big \rangle$. Thus, assuming that $\{A_1,A_2,A_3\}$ meet the mutual overlap constraint, $\texttt {form}(A_1,A_2,A_3)$ is the primary diagram:

**Diagram 13:**
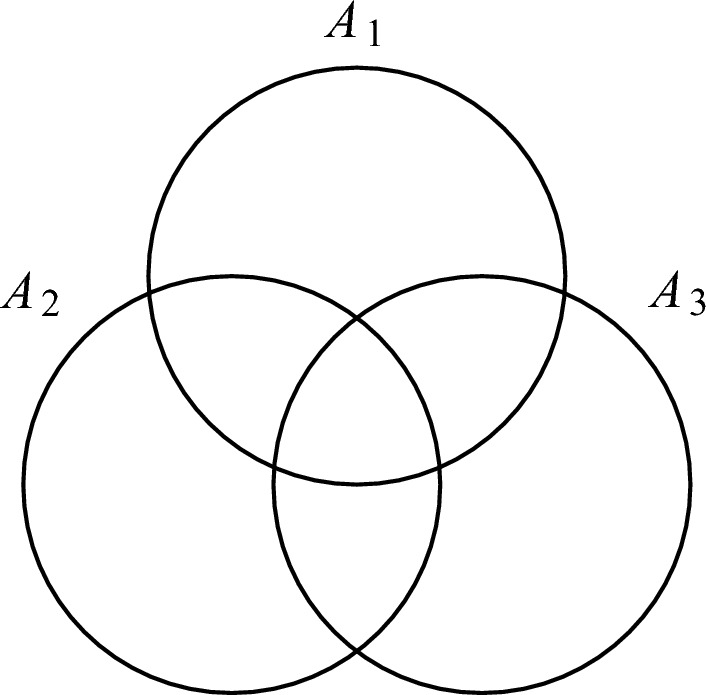
Primary Venn diagram

**Diagram 14:**
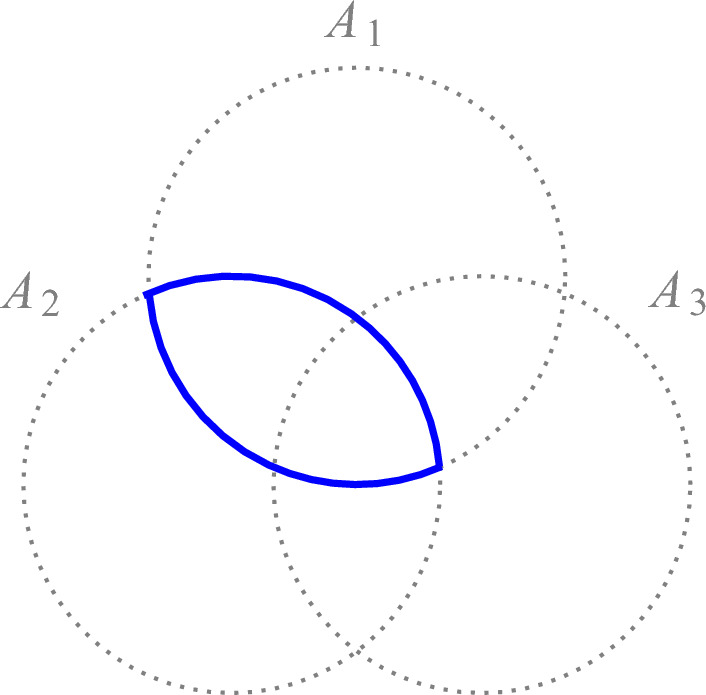
$$\texttt {inter}(A_1,A_2)$$

**Diagram 15:**
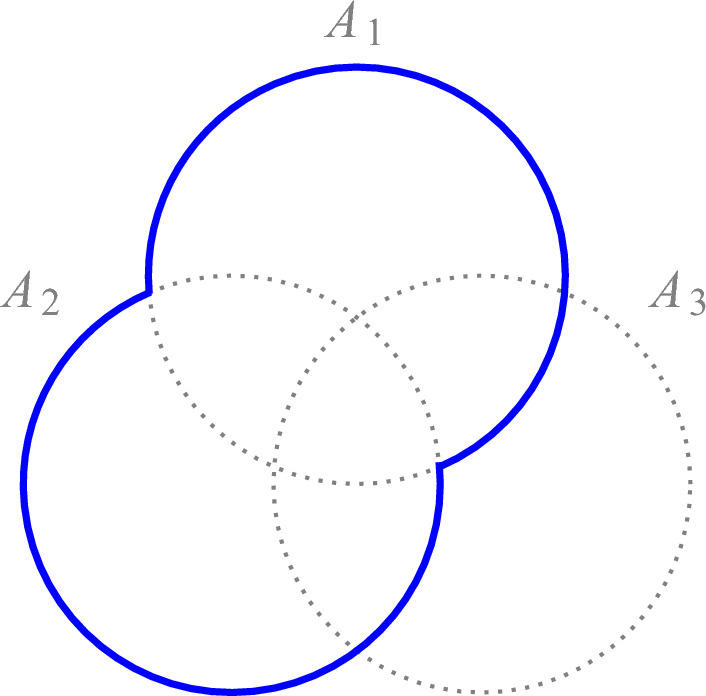
$$\texttt {union}(A_1,A_2)$$

Next, to decorate a diagram, we first need rules for constructing regions of a diagram out of the basic regions and background. The regions of a diagram include its basic regions and the background *R*. Further regions can be specified as non-empty intersections, unions, and relative complements of these regions. Thus, the syntax will have three operations for forming regions out of other regions $\texttt {inter}$, $\texttt {union}$, and $\texttt {comp}$. Given two regions $X_1$ and $X_2$ of a diagram *D*, $\texttt {inter}(X_1,X_2)$ = $\bigcap \{X_1,X_2\}$, $\texttt {union}(X_1,X_2)$ = $\bigcup \{X_1,X_2\}$, and $\texttt {comp}(X_1,X_2)$ = $X_1 - X_2$ are regions of *D* provided that they are non-empty. The resulting regions are just as one would expect, for example Diagrams 13, 14 and 15:

**Diagram 16:**
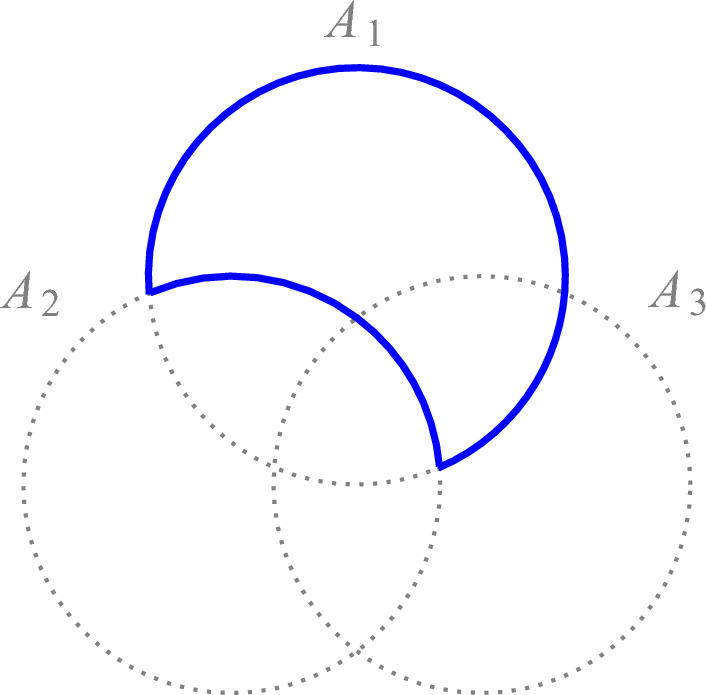
$$\texttt {comp}(A_1,A_2)$$

Given a diagram *D* and region *X* in *D*, the diagram can be decorated by destroying or saving the region *X*. The syntax rule $\texttt {destroy}$ applied to a diagram $\langle B, \Delta , \Sigma \rangle$, and one of its regions *X* results in expanding the destroyed region Δ with *X*. Thus, if $D = \langle B, \Delta , \Sigma \rangle$, then $\texttt {destroy}(D,X)= \langle B, \Delta \cup X, \Sigma \rangle$. So, $\texttt {destroy}$ applied to Diagram 12 and the region $\texttt {inter}(A_1,A_2)$ results in Diagram 3. That is, Diagram 3 is constructed as follows:

$$\texttt {destroy}\big (\texttt {form}(A_1,A_2,A_3)$$,$$\texttt {inter}(A_1,A_2)\big )$$ = $$\big \langle \{A_1,A_2,A_3\}, A_1\cap A_2, \emptyset \big \rangle $$.

The salvation rule, $\texttt {save}$, applied to a diagram $\langle B, \Delta , \Sigma \rangle$ and one of its regions *X* adds *X* to the set of saved regions Σ. Thus, if $D=\langle B, \Delta , \Sigma \rangle$, then $\texttt {save}(D,X) = \langle B, \Delta , \Sigma \cup \{X\} \rangle$. So, $\texttt {save}$ applied to Diagram 3 (i.e., $\langle \{A_1,A_2,A_3\}, A_1\cap A_2, \emptyset$) and the region $\texttt {comp}(A_3, A_1)$ results in Diagram 16:

**Diagram 17:**
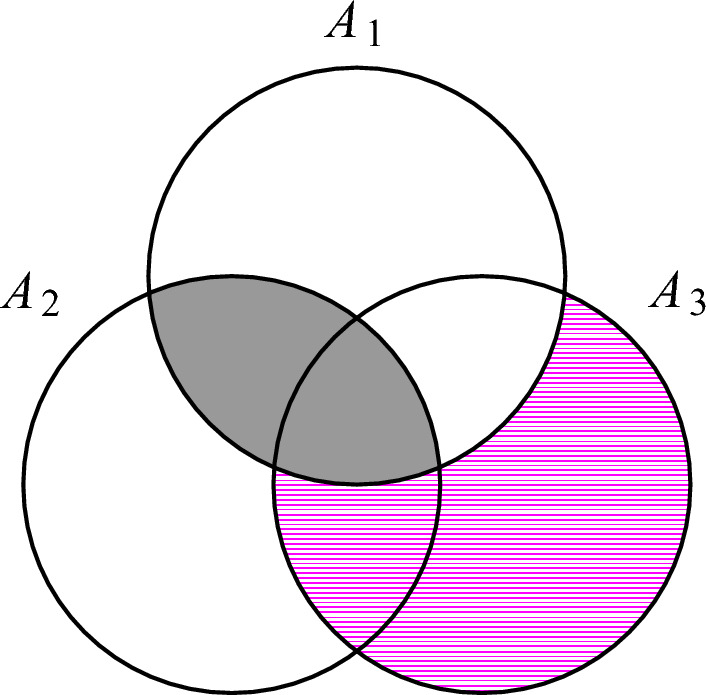
$$\bigg \langle \big \{A_1,A_2,A_3\big \}, A_1\,{\cap }\,A_2,\,\big \{ A_3\,{-}\,A_1\big \}\bigg \rangle $$

We could further build on this by saving another region. For example, to save the region $X= \texttt {comp}(A_1,\texttt {union}(A_2,A_3))$ we could apply $\texttt {save}(\text {Diagram} 16,X)$ to get Diagram 17:

**Diagram 18:**
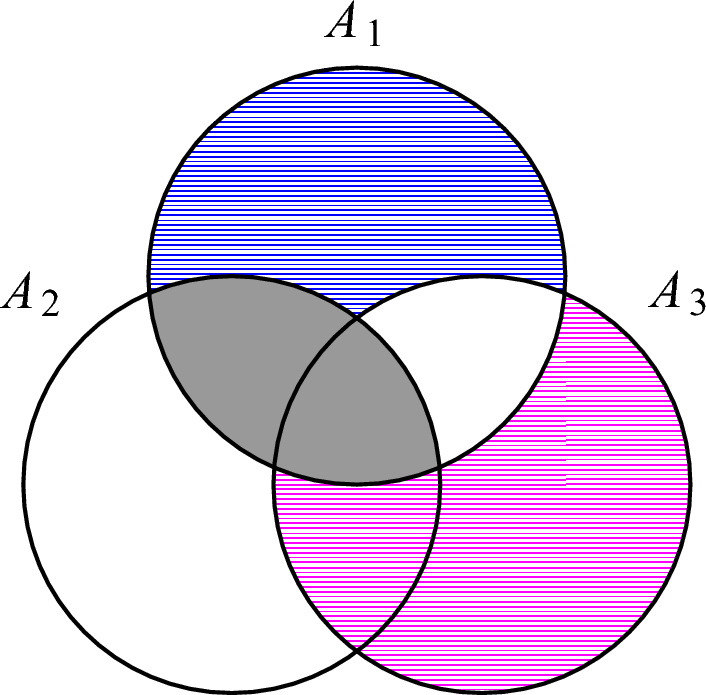
$$\bigg \langle \big \{A_1,A_2,A_3\big \}, A_1\cap A_2, \big \{A_3 -A_1, A_1 - (A_2 \cup A_3)\big \} \bigg \rangle $$

Thus, the region-first syntax for Venn diagrams can be summarized as follows. Every simple closed curve in the entire plane *R* determines a region bounded by the curve. We call these regions the *basic regions*. Thus the atomic representational elements of our syntax are drawn from this set:

- **Basic Regions**: $$\{A_1, A_2,\ldots , A_i,\ldots \} $$

Based on this, all the Venn diagrams can be constructed via the six syntactic construction rules summarized as follows:

- **Form Rule**: If $X_1,\ldots X_n$ are basic regions satisfying the mutual overlap constraint, then $\texttt {form}(X_1,\ldots , X_n)$ is a diagram with regions including $X_1,\ldots , X_n$ and *R*.
- **Intersection Rule**: If $X_1$ and $X_2$ are regions of *D*, then $\texttt {inter}(X_1,X_2) = \bigcap \{X_1,X_2\}$ is a region of *D*, provided that it is non-empty.
- **Union Rule**: If $X_1$ and $X_2$ are regions of *D*, then $\texttt {union}(X_1,X_2) = \bigcup \{X_1,X_2\}$ is a region of *D*, provided that it is non-empty.
- **Complement Rule**: If $X_1$ and $X_2$ are regions of *D*, then $\texttt {comp}(X_1,X_2) = X_1 - X_2$ is a region of *D*, provided that it is non-empty.
- **Destruction Rule**: If *D* is a diagram and if *X* is a region of *D*, then $\texttt {destroy}(D,X)$ is a diagram.
- **Salvation Rule**: If *D* is a diagram and if *X* is a region of *D*, then $\texttt {save}(D,X)$ is a diagram.

For example this is a syntactic derivation of Diagram 3:^14^

**Figure:**
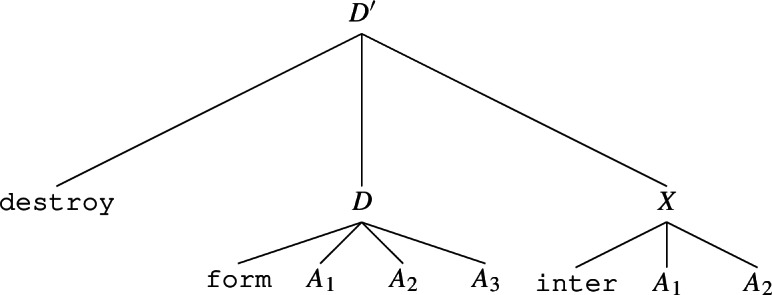

### The region-first semantics

We have specified rules for syntactically deriving each Venn diagram from a set of basic regions. The *Form Rule* can be used to derive a diagram out of a set of basic regions. The *Intersection*, *Union*, and *Complement Rules* can be used to derive a new region of a diagram from a pair of its regions. The *Destruction Rule* and *Salvation Rule* can be used to derive a new diagram from a diagram and one of its regions.

A compositional semantics for Venn diagrams would assign semantic values to the atomic representations (the basic regions) and semantic evaluation rules for determining the semantic value of a complex representation (such as a non-basic region or diagram) from the semantic values assigned to the simpler representations from which it is derived. We will use the function $\llbracket .\rrbracket _\mathfrak {A}$ to represent the semantic value of a representation relative to model $\mathfrak {A}$. A model $\mathfrak {A}$ is a pair $\langle U, I\rangle$, where *U* is a universe (or domain of individuals) and *I* is an interpretation function which maps basic regions to subsets of the universe.

The assignment of semantic values to complex representations will take two forms corresponding to the types of complex representations. In this case, the first task is to specify rules determining the semantic values of the nonbasic regions in terms of the simpler regions from which they are derived. The second task is to specify the rules determining the semantic value of a primary diagram from semantic values of the basic regions that form it and then to specify the semantic values of complex diagrams in terms of the diagrams from which they are derived.

#### Semantics for regions

The basic vocabulary for regions consists of the basic regions $X_1, X_2,\ldots$ and the background *R*. As we said above, a model $\mathfrak {A}$ is a pair consisting of a universe of discourse *U* and an assignment *I*. Relative to any model the background region *R* represents the entire universe, while each basic region represents some subset of the universe. Thus the semantic value of *R* will just be the universe:

- For the background region *R*, $\llbracket R \rrbracket _\mathfrak {A} = U$, where $\mathfrak {A} = \langle U,I\rangle$.

And the semantic value of a basic region *X* will be its value under *I*.

- For any basic region *X*, $\llbracket X \rrbracket _\mathfrak {A} = I(X)$, where $\mathfrak {A} = \langle U,I\rangle$.

The semantic value of a derived region will be determined as a function of the regions from which it is derived in the ordinary way. The intersection of two regions will represent the intersection of the sets represented by each region, the union of two regions will represent the union of two sets represented by those regions, and the relative complement of two regions will represent the corresponding relative complement of the sets they represent. More formally:

- $$\llbracket \texttt {inter}(X_1,X_2) \rrbracket _\mathfrak {A} = \llbracket X_1\rrbracket _\mathfrak {A} \cap \llbracket X_2\rrbracket _\mathfrak {A}$$
- $$\llbracket \texttt {union}(X_1,X_2)\rrbracket _\mathfrak {A} = \llbracket X_1\rrbracket _\mathfrak {A} \cup \llbracket X_2\rrbracket _\mathfrak {A}$$
- $$\llbracket \texttt {comp}(X_1,X_2)\rrbracket _\mathfrak {A}= \llbracket X_1\rrbracket _\mathfrak {A} - \llbracket X_2\rrbracket _\mathfrak {A}$$

The semantics thus far is not essentially different from the standard semantics (Shin, 1994; 65, Hammer & Danner, 1996: 470-1).

#### Semantics for diagrams

Turning now to the semantics for diagrams, we will first specify the semantic value of a diagram *D* that results from applying the rule $\texttt {form}$ to a set of basic regions $X_1,\ldots , X_n$. We will then specify how to derive new diagrams from old diagrams by applying the rules $\texttt {destroy}$ and $\texttt {save}$. It is at this stage that the semantic theory offered by Shin (1994) and Hammer and Danner (1996) is not compositional.

A primary diagram is true on any model.^15^ Therefore, if a diagram *D* is the result of applying the form rule to basic regions $X_1,\ldots ,X_n$, then *D* will be true on any model. Thus, the semantic rule for $\texttt {form}$ is trivial.

- $$\llbracket \texttt {form}(X_1,\ldots ,X_n)\rrbracket _\mathfrak {A}$$ = 1.

Decorated diagrams derive from applying the rules $\texttt {destroy}$ and $\texttt {save}$ to pairs consisting of a simpler diagram and a region. The syntactic rules $\texttt {destroy}$ and $\texttt {save}$ each take a diagram *D* and a region *X* in *D* to yield a diagram $D^{\prime }$. The semantic rule corresponding to $\texttt {destroy}$ says that $D^{\prime }$ will be true in a model if *D* is true in that model and *X* is empty.

- $$\llbracket \texttt {destroy}(D,X) \rrbracket _\mathfrak {A} = {\left\{ \begin{array}{ll} 1, \textit{if } \llbracket D \rrbracket _\mathfrak {A} = 1 \; \textit{and } \; \llbracket X\rrbracket _\mathfrak {A}= \emptyset , \\ 0, \textit{otherwise.} \end{array}\right. }$$

Similarly, the semantic rule corresponding to save says that $D^{\prime }$ will be true in a model if *D* is true in that model and *X* is non-empty.

- $$\llbracket \texttt {save}(D,X) \rrbracket _\mathfrak {A} = {\left\{ \begin{array}{ll} 1, \textit{if } \llbracket D \rrbracket _\mathfrak {A} = 1 \; \textit{and } \; \llbracket X\rrbracket _\mathfrak {A}\ne \emptyset ,\\ 0, \textit{otherwise.} \end{array}\right. }$$

The truth-conditions of a diagram such as Diagram 3 = $\texttt {destroy}\big (\texttt {form}(A_1,A_2,A_3)$, $\texttt {inter}(A_1,A_2)\big )$ can then be derived as follows.^16^

**Figure:**
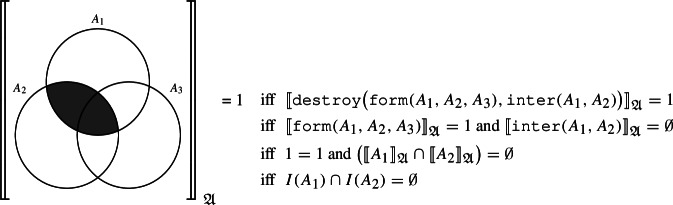

## The cell-first approach

We turn now to the *cell-first* approach. Both approaches form a diagram out of a set of basic regions. They differ in how they decorate the diagram. The region-first approach identified certain regions of the diagram by sequentially applying set-theoretic union, intersection, and relative complement to the basic regions. These regions could then be decorated by the rules destroy or save.

In the cell-first approach, the diagram is decorated by first identifying *cells* or minimal regions in terms of the basic regions. The cells can then be decorated by applying the rules destroy or save. In the cell-first approach, the rule save will complicate both the syntax and the semantics because we will need to distinguish the case in which a collection of cells each represent a non-empty set from the case in which a collection of cells *collectively* represent a non-empty set.

As we have mentioned, both Shin (1994) and Hammer and Danner (1996) “mix-and-match”, whereby they offer region-first rules for some constructions, but cell-first rules for others. The important thing to note is that they both construct cells and offer some cell-first syntactic construction and semantic evaluation rules.^17^ But, as with the region-first approach, these rules are not specified compositionally. Our syntax and semantic rules discussed below are specified compositionally.

### The cell-first syntax

In order to accommodate changes in the save rule, we will need to slightly modify our definition of a diagram. For the cell-first approach, in a Diagram $D = \langle B, \Delta ,\Sigma \rangle$, the second component Δ and the third component Σ will be modified. First, rather than being a region, Δ will now be modeled as a set of “destroyed” cells. Second, rather than a set of regions, Σ will be a set of pairs. Each pair will include a natural number and a cell. The set of cells paired with the same number in Σ is “collectively” saved.

As with the region-first approach, the cell-first approach begins with the rule form which forms a primary diagram *D* from a collection of basic regions. This rule is defined as before.

- **Form Rule**: If $A_1,\ldots ,A_n$ are regions satisfying the mutual overlap constraint, then $\texttt {form}(A_1,\ldots ,A_n)$ = $\langle \{A_1,\ldots ,A_n\}, \emptyset , \emptyset \rangle$ is a diagram.

The diagram *D* will then be decorated in terms of its cells.

We will call the rule for identifying cells ‘$\texttt {cell}$’. This rule takes basic regions of a diagram in two sequences as its arguments. It identifies a cell in terms of the relative complement of the intersection of one sequence (and the plane) and the union of the other. That is:

- **Cell Rule**: If $\{X_1,\ldots ,X_j\} \cup \{Y_1,\ldots ,Y_k\}$ = $D_B$, then $\texttt {cell}_{ Y_1,\ldots ,Y_k} (X_1,\ldots ,X_j)$ is a cell of diagram *D*, where $\texttt {cell}_{ Y_1,\ldots ,Y_k} (X_1,\ldots ,X_j)$ = $\bigcap \big \{ R, X_1,\ldots ,X_j\} - \bigcup \big \{Y_1,\ldots ,Y_k\big \}$, and where *R* is the total plane.^18^

In Diagram D = $\texttt {form}(A_1,A_2,A_3)$, each region *a*-*h* can be identified through the $\texttt {cell}$ operator. Thus, region *c* is $\texttt {cell}_\emptyset (A_1,A_2,A_3)$, region *a* is $\texttt {cell}_{A_2,A_3}(A_1)$, region *d* is $\texttt {cell}_{A_2,}(A_1,A_3)$, and so on.

**Figure:**
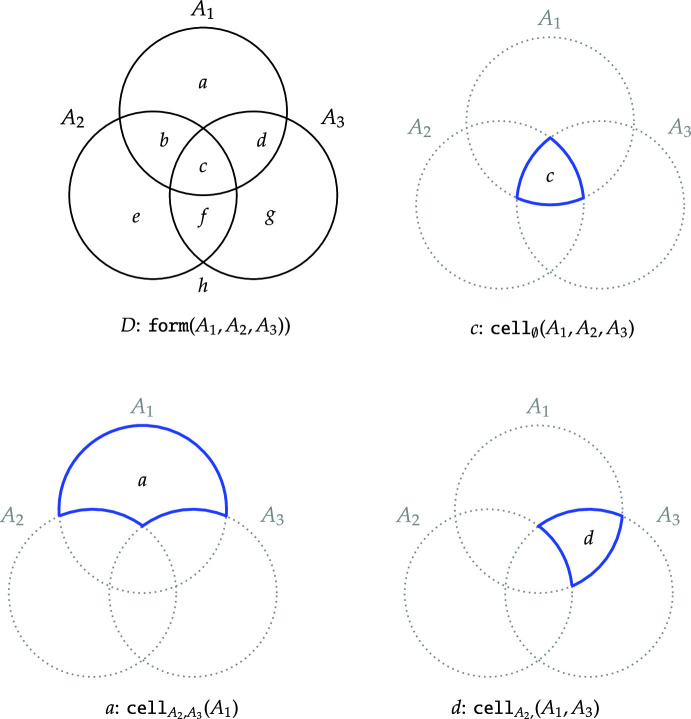

The cells of a diagram can then be used to decorate it following the destroy and save rules.

The rule destroy functions just as it did on the region-first approach, except that it takes a cell rather than an arbitrary region as argument. That is, the rule takes a diagram and a cell as arguments. It returns a diagram in which the destroyed region now includes that cell. That is,

- **Destruction Rule**: If $D = \langle B, \Delta , \Sigma \rangle$ is a diagram and *X* is a cell of *D*, then $\texttt {destroy}(D,X)= \langle B, \Delta \cup \{ X\}, \Sigma \rangle$.

A destroyed cell will be represented by shading just as before.

Turning now to the rule $\texttt {save}$, the cell-first approach will need to revise how this rule operates. As we have seen, we need to distinguish the case in which multiple cells are each non-empty from the case in which multiple cells are collectively non-empty. Because the cell-first approach works on a cell-by-cell basis, we will need some procedure to identify and extend a saved region. Venn proposed to do this by decorating a diagram with a number. All of the cells in a diagram that are decorated with the same number are *collectively* saved.

It follows that our save rule will take *three* arguments: a diagram, a cell of that diagram, and a number *n*. The output of this rule changes the sequence of saved regions of the diagram by expanding the *n*th member of this sequence to also include the cell.

- **Salvation Rule**: If $D = \langle B, \Delta , \Sigma \rangle$ is a diagram, *X* is a cell of *D*, and $i \in \mathbb {N}$, then $\texttt {save}(D,X,i)= \big \langle B, \Delta , \Sigma \cup \{ \langle i, X\rangle \} \big \rangle$.

Thus, instead of Diagram 17, which is what we derived on the region-first syntax, we have Diagram 18:

**Diagram 19:**
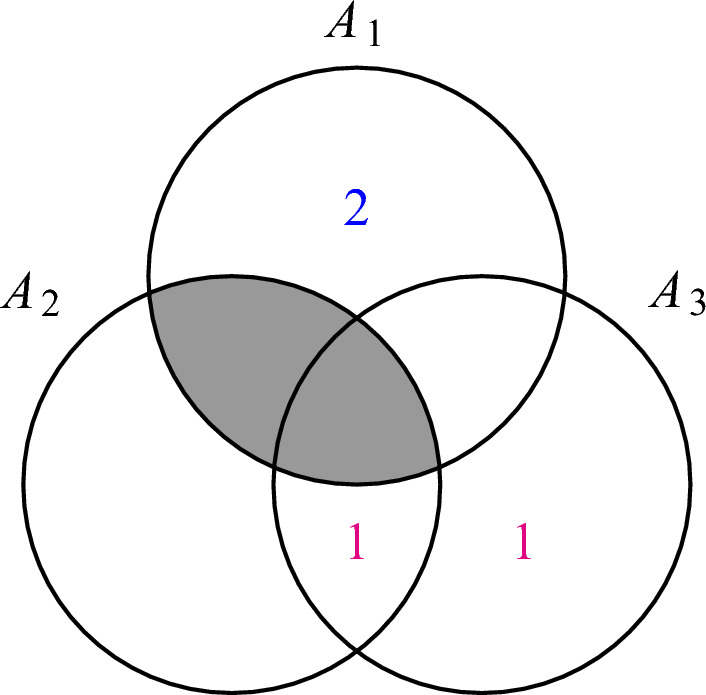

The full syntactic derivation of Diagram 18 is in Appendix A.

### The cell-first semantics

The cell-first semantics differs from the region-first semantics in two important ways. First, rather than offering a rule for semantically evaluating regions in terms of the regions from which they derive, the cell-first semantics has a rule for directly evaluating cells.

- $$\llbracket \texttt {cell}_{ Y_1,\ldots ,Y_k} (X_1,\ldots ,X_j)\rrbracket _\mathfrak {A} = \bigcap \big \{U,\llbracket X_1\rrbracket _\mathfrak {A},\ldots ,\llbracket X_j\rrbracket _\mathfrak {A} \big \} - \bigcup \big \{\llbracket Y_1\rrbracket _\mathfrak {A},\ldots ,\llbracket Y_k\rrbracket _\mathfrak {A}\big \}$$

According to this rule, a cell denotes a subset of the domain that is included in the subsets represented by $X_1,\ldots ,X_j$ and which overlaps with none of the subsets represented by $Y_1,\ldots ,Y_k$.

Secondly, the cell-first semantics will be more complicated than the region-first semantics because of the differences in how the approaches handle “salvation”. On both approaches the semantic evaluation rule for destroy is more straightforward. The rule destroy takes a diagram *D* to a new diagram $D^{\prime }$. If $D^{\prime }$ is true in a model, then *D* must be true in that model as well. Applying destroy to another region to yield another diagram would further restrict the set of models in which the resulting diagram is true.

**Diagram 20:**
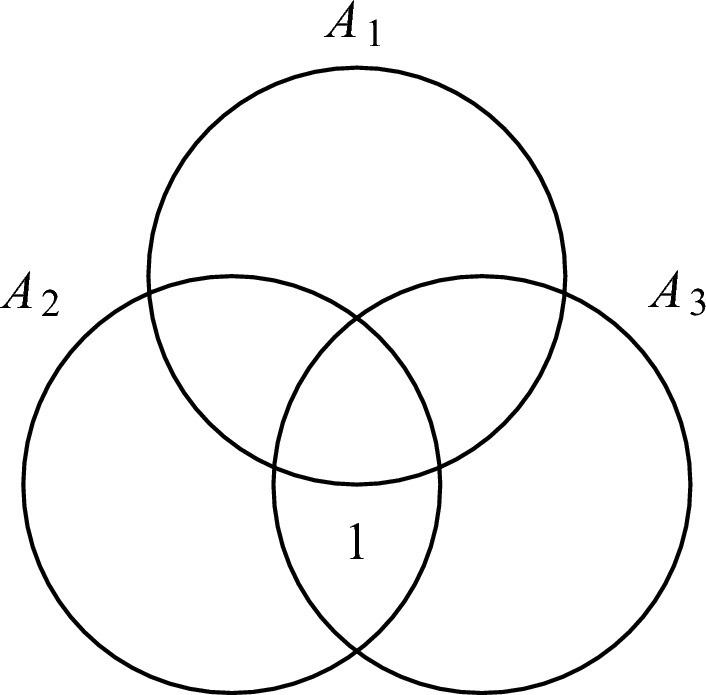
$$\Sigma : \bigg \{\big \langle 1, (A_2\,{\cap }\,A_3)\,{-}\,A_1\big \rangle \bigg \}$$

In contrast, save takes a diagram *D* to a new diagram $D^{\prime }$ that may be true in *more models*. In this sense, the result of applying save to a diagram loses information. For example, consider the derivation of Diagram 20 which results from adding the number 1 to cell $$g = A_3 -(A_1 \cup A_2)$$ of Diagram 19.

**Diagram 21:**
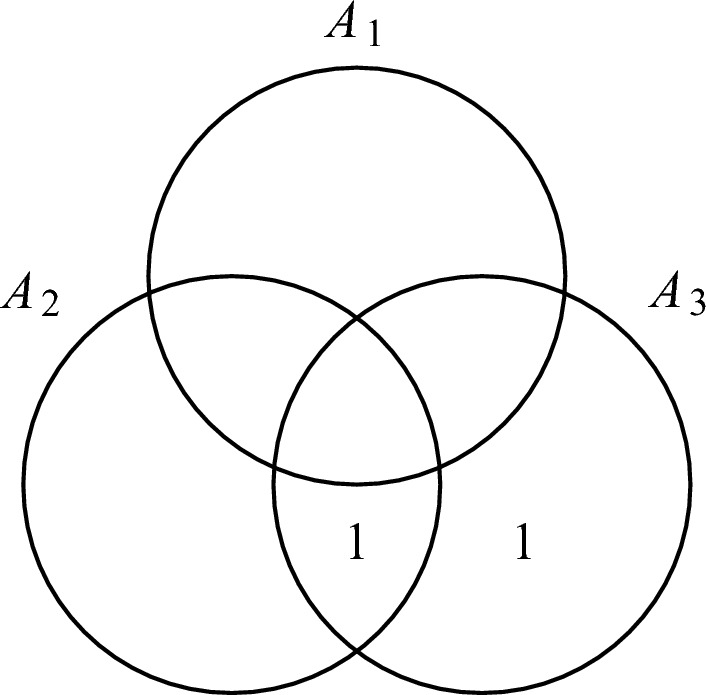
$$\Sigma : \bigg \{ \big \langle 1, (A_2\cap A_3) -A_1\big \rangle , \big \langle 1, A_3 -(A_1 \cup A_2)\big \rangle \bigg \}$$

Diagram 19 already has the variable 1 in cell $f= (A_2\cap A_3) -A_1$. Thus, 19 is true only in models in which cell *f* denotes a non-empty set. Diagram 20, however, does not require cell *f* to denote a non-empty set. Rather, Diagram 20 requires the union of the sets denoted by *f* and *g* to be non-empty. To paraphrase Venn, destruction is conjunctive whereas salvation is only disjunctive. This difference requires us to assume more semantic structure in a diagram in order to explain how the semantic value of Diagram 20 depends on the semantic value of Diagram 19.

The problem in essence is the following. We begin with a diagram that is true in a certain class of models. The diagram may already contain occurrences of the number *n* in certain cells. This means that the diagram is true only in models in which the union of the denotations of these cells—call it *Y*—is non-empty. A diagram that adds number *n* to a new cell *X* will be true in some models in which the denotation of *Y* is empty so long as the denotation of the new cell *X* is non-empty. Thus, in the semantics, we need to keep track of which cells have variable chains in them in addition to tracking constraints on the model.

To solve the problem, we introduce a new parameter in our semantics, the “existential assignment” parameter. Intuitively, this parameter will track the *saved* regions—the regions in which something must exist. This will be captured by letting each existential assignment σ be a set of pairs $\langle i, z\rangle$, where *i* is a natural number and *z* a subset of *U* (where *z* is assigned to some cell *X*).^19^ The basic idea is that the syntactic rule $\texttt {save}$ adds a number to a cell of a diagram. The existential assignment will keep track of which subsets of the domain are associated with which numbers. The semantic function of adding a number *i* to cell *X* in a diagram *D* through the rule save is to add the pair consisting of *i* and the denotation of *X* (or, $\langle i, \llbracket X\rrbracket _\mathfrak {A}\rangle )$ to the existential assignment.

For any model $\mathfrak {A}$ and assignment σ, we will first provide a definition of $\llbracket .\rrbracket _\mathfrak {A}^\sigma$. Once we have given a semantics on which any diagram *D* will be true or false relative to an existential assignment, we will need to extract absolute de-relativized truth conditions for *D*. To do this, we will define truth for a diagram as truth relative to the null existential assignment. That is, a diagram *D* is *true* in a model $\mathfrak {A}$ iff $\llbracket D \rrbracket _\mathfrak {A}^\emptyset = 1$.^20^

The existential assignment σ will enter the semantics through the interpretation of the rule $\texttt {form}$. In our revised semantics, the result of applying $\texttt {form}$ to basic regions $X_1$,...,$X_n$ will be true *relative to an existential assignment* σ if and only if the regions indexed by numbers in σ are all non-empty.

Thus, each diagram will be true or false only relative to both a model and an existential assignment. The rule save applied to diagram *D*, cell *X*, and number *i* updates the existential assignment σ and model $\mathfrak {A}$ at which *D* is evaluated by adding the pair $\langle i, \llbracket X\rrbracket ^\sigma _\mathfrak {A}\rangle$ to σ. That is, the updated assignment $\sigma ^{\prime }$ will be as follows:

$$\sigma ^{\prime } = \sigma \cup \big \{ \big \langle i, \llbracket X\rrbracket ^\sigma _\mathfrak {A}\big \rangle \big \}$$

Given any existential assignment σ, we will need to extract the union of the sets assigned to some number *i*, as follows:

#### Definition 2

For any existential assignment σ, let $\sigma _i = \bigcup _{\langle i, z\rangle \in \sigma } z$.

Using these resources, we are now in a position to fully state the semantics for Venn diagrams including existential information. Let a model for our system of Venn diagrams be a pair $\mathfrak {A}=\langle U,I \rangle$, where *U* is a set of objects and *I* is an interpretation of the basic regions—it maps basic regions to subsets of *U*.

  **Clause 1.** For the background region *R*, $\llbracket R \rrbracket _\mathfrak {A} = U$,

  **Clause 2.** For any basic region *X*, $\llbracket X\rrbracket ^\sigma _\mathfrak {A} = I(X)$,

  **Clause 3.** For any number $i\in \mathbb {N}$, $\llbracket i\rrbracket ^\sigma _\mathfrak {A}= \sigma _i$

  **Clause 4.** $$\llbracket \texttt {form}(X_1,\dots ,X_n)\rrbracket ^\sigma _\mathfrak {A}\ = {\left\{ \begin{array}{ll}1, \text {if } \sigma _i \ne \emptyset , \text { for all }i \in dom(\sigma ), \\ 0, \textit{otherwise.} \end{array}\right. }$$

  **Clause 5.** $$\llbracket \texttt {cell}_{ Y_1,\ldots ,Y_k} (X_1,\ldots ,X_j)\rrbracket ^\sigma _\mathfrak {A} = \bigcap \big \{U,\llbracket X_1\rrbracket ^\sigma _\mathfrak {A},\ldots ,\llbracket X_j\rrbracket ^\sigma _\mathfrak {A} \big \} - \bigcup \big \{\llbracket Y_1\rrbracket ^\sigma _\mathfrak {A},\ldots ,\llbracket Y_k\rrbracket ^\sigma _\mathfrak {A}\big \}$$

  **Clause 6.** $$\llbracket \texttt {destroy}(D,X) \rrbracket ^\sigma _\mathfrak {A} = {\left\{ \begin{array}{ll} 1, \textit{if } \llbracket D \rrbracket ^\sigma _\mathfrak {A} = 1 \text { and } \llbracket X\rrbracket ^\sigma _\mathfrak {A}= \emptyset , \\ 0, \textit{otherwise.} \end{array}\right. }$$

  **Clause 7.**$$\llbracket \texttt {save}(D,X,i) \rrbracket ^\sigma _\mathfrak {A} ={\left\{ \begin{array}{ll} 1,\textit{if } \llbracket D \rrbracket ^{\sigma ^\prime }_\mathfrak {A} = 1, \text { where } \sigma ^{\prime } = \sigma \cup \big \{ \big \langle i, \llbracket X\rrbracket ^\sigma _\mathfrak {A}\big \rangle \big \}, \\ 0, \textit{otherwise.} \end{array}\right. }$$

To see the semantics applied to a diagram see appendix A, where there is a detailed walk-through applied to Diagram 18.

There is one lingering non-compositional aspect to the presentation above. We have specified semantic values of diagrams *relative to salvation sequences*. The truth-value of a diagram relative to one of these sequences may depend on the truth-value of a constituent diagram *relative to a different sequence*. To make the semantics fully compositional, one would have to treat the semantic value of a diagram as a function from salvation sequences into truth-values. This would be analogous to treating the semantic value of a formula of first-order logic as a function from assignment functions (or sequences) into truth-values.^21^

## Peirce diagrams

**Diagram 22:**
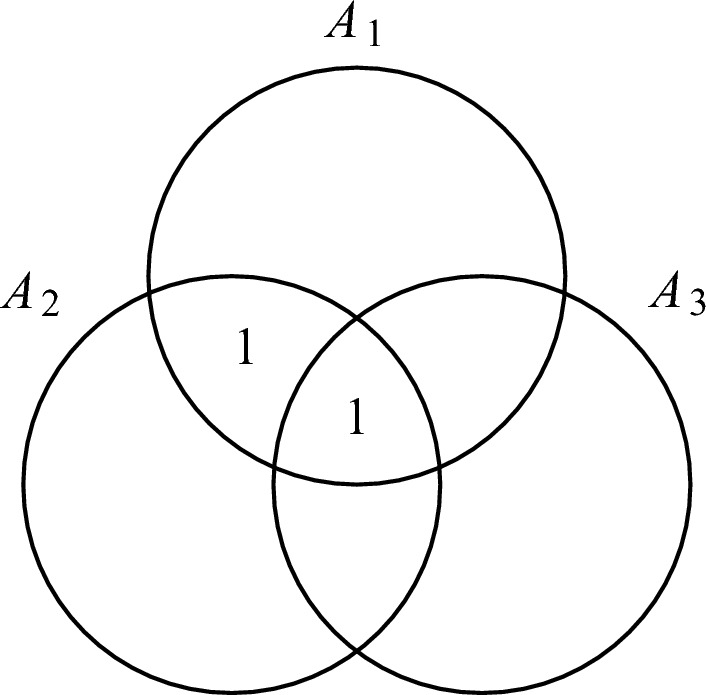
.

The cell-first diagrams contain more structure than the region-first diagrams because the saved regions are modeled by pairs of numbers and cells. The number plays a role in both the syntactic derivation and semantic evaluation of the diagram. Essentially, each number designates a function from the existential assignment sequence to a subset of the domain. Distinct numbers will be associated with different such functions. Thus, the numbers 1 and 2 have different denotations. This difference in denotation is required to guarantee that, for example, Diagrams. 21 and 22 have different truth conditions.

**Diagram 23:**
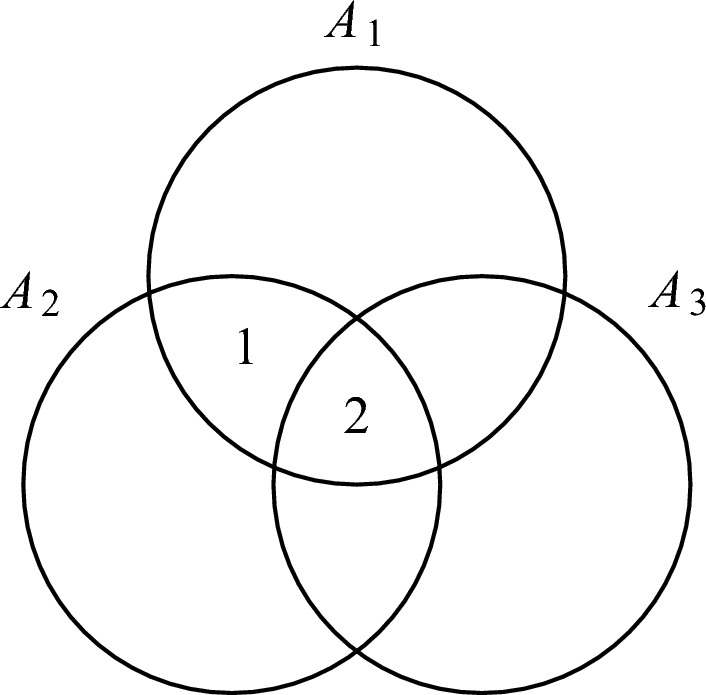
.

**Diagram 24:**
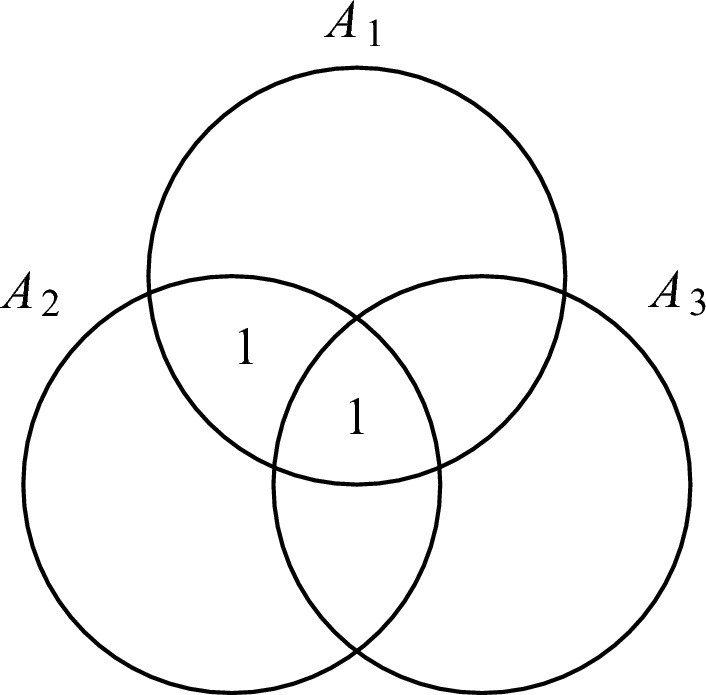
.

Yet, one might worry about a certain inelegance in this approach. Just as is the case in first-order logic, where one might think that alphabetic variants—$\forall x_1 (x_1 = x_1)$ and $\forall x_2 (x_2 = x_2)$—are synonymous, it is tempting to view Venn diagrams that are “alphabetic variants” of each other as synonymous. Thus, Diagrams. 23 and 24 seem to be mere re-labelings of one another.

**Diagram 25:**
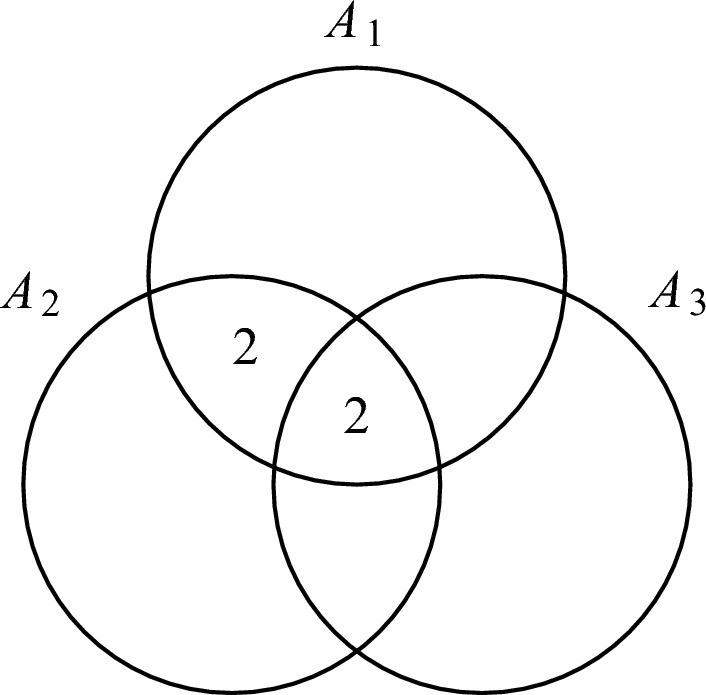
.

Thus, distinct diagrams seem to represent the same set of models. This sort of concern might lead one to prefer the Peirce Diagram 25 as representing what is common to Diagrams 23 and 24.

**Diagram 26:**
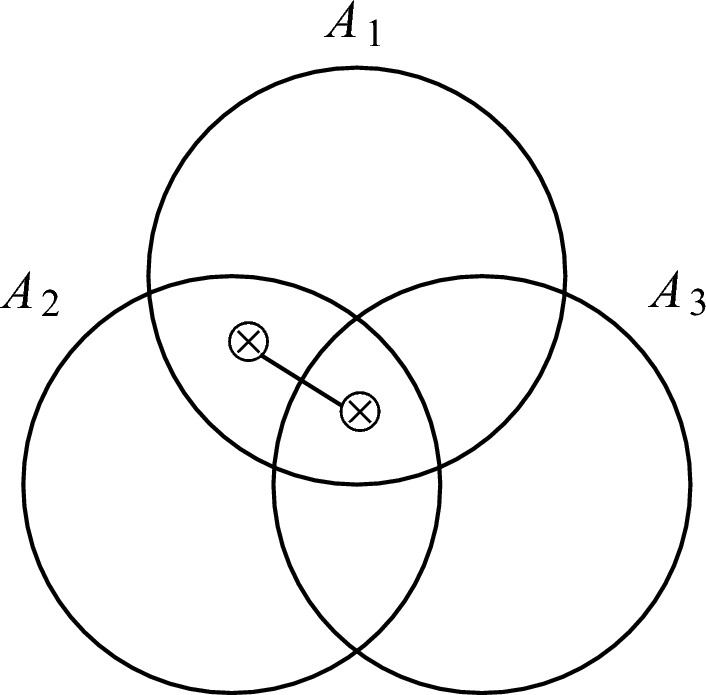
.

For instance, Moktefi and Pietarinen (2015) cite the move to Peirce diagrams as an improvement for this reason.

The ‘connecting-line’ convention clearly improves Venn’s numbering method. Instead of different numbers for the same information (existence), Peirce uses a unique convention for existentials and secures alternatives with the connecting lines. (Moktefi & Pietarinen, 2015: 370)

There is an analogous tradition in logic associated with Quine and Bourbaki, whereby one specifies a quantified formula with a “wire diagram”, where explicit variables are not found in the typography (see Quine, (1940: 69-70) and Bourbaki, 1954).^22^

We suggest that Peirce diagrams can be reconstructed as the result of applying a transformation rule to numerically indexed Venn diagrams. In particular, a Peirce diagram, logically speaking, should be viewed as an equivalence class of alphabetic variant Venn diagrams. First, we define the notion of alphabetic variant Venn diagrams.

- Diagrams $D_1$ = $\langle B, \Sigma , \Delta _1\rangle$ and $D_2$ = $\langle B, \Sigma , \Delta _2\rangle$ are alphabetic variants if and only if for some injective $f: \mathbb {N} \longrightarrow \mathbb {N}$, $\Delta _2$ = $\{\langle f(n),S\rangle : \langle n,S\rangle \in \Delta _1 \}$

A transformation rule will be a syntactic rule that takes a Venn diagram to a Peirce Diagram, which will be formally implemented as an equivalence class of alphabetic variant Venn diagrams (cf. Lewis, 1970: 45-6). The issue bears resemblance to problems connected to the “antinomy of the variable” in first-order logic.^23^

## Compositionality and the standard semantics

Recall that a semantics is compositional if for every representation *E* constructed by applying syntactic formation rule *R* to constituents $\beta _1,\ldots ,\beta _n$, there is a function $f_R$ such that the meaning of *E* is the result of applying the function *f* to the meanings of $\beta _1,\ldots ,\beta _n$. The two semantic theories we have offered are each explicitly compositional in that every semantic evaluation rule is framed as an assignment of meaning to a diagram in terms of the application of a function to its constituent representations. In our semantics, if a diagram *D* derives from representations $\beta _1,\ldots ,\beta _n$, then the corresponding semantic compositional rule will specify the semantic value of *D* by applying a function to the semantic values of $\beta _1,\ldots ,\beta _n$.

In contrast, the standard semantics of Shin (1994) and Hammer and Danner (1996) is not framed to be compositional. Both Shin and Hammer and Danner offer syntactic theories that specify each diagram *D* in terms of some syntactic rule applied to constituent representations $\beta _1,\ldots ,\beta _n$. However, in neither case do they specify the meaning or semantic value of the diagram by applying a function (specified by the syntactic construction rule) to the semantic values of $\beta _1,\ldots ,\beta _n$. Rather, both Shin and Hammer and Danner simply appeal to the final outputs of the syntax rules. In our framework, these correspond to the $\langle B, \Delta , \Sigma \rangle$ triples. Shin (1994) and Hammer and Danner (1996) each provide rules specifying the truth conditions of diagrams in terms of these triples (or equivalent representations).^24^ Thus, semantic theories in these works are not rule-to-rule compositional. In this section, we will briefly focus on the semantics of Shin (1994) and show that it cannot easily be made compositional.

The syntactic derivations offered by Shin (1994) and by Hammer and Danner (1996) differ from our own (and from each other) by “mixing and matching” region and cell first rules. The syntax of Shin, (1994, 57) offers a broadly “region-first” syntax for destruction but a “cell-first” syntax for salvation. Shin’s semantics for salvation proceeds in two stages, first by introducing a variable *x* into a cell and then by uniting this variable with an existing chain. To simplify, we will assimilate this syntax to the cell-first syntax above, with the syntactic rules as follows:

- **Destruction Rule**: If $D = \langle B, \Delta , \Sigma \rangle$ is a diagram and *X* is a cell of *D*, then $\texttt {destroy}(D,X)= \langle B, \Delta \cup \{ X\}, \Sigma \rangle$.
- **Salvation Rule**: If $D = \langle B, \Delta , \Sigma \rangle$ is a diagram, *X* is a cell of *D*, and $i \in \mathbb {N}$, then $\texttt {save}(D,X,i)= \big \langle B, \Delta , \Sigma \cup \{ \langle i, X\rangle \} \big \rangle$.

Shin’s semantics then effectively specifies the denotation of a diagram *D* in terms of the triple $\langle B, \Delta , \Sigma \rangle$ rather than in terms of the meanings of the representations that went into syntactically deriving it. Shin’s (1994, 69-71) semantics can be re-written in our notation as follows:

- For a diagram *D* (specified by a $\langle B, \Delta , \Sigma \rangle$ triple) relative to a model $\mathfrak {A}$, $\llbracket D \rrbracket _\mathfrak {A} = 1$ iff
  (i) $$\llbracket \Delta \rrbracket _\mathfrak {A} = \emptyset $$, and
  (ii) for every *i*, $\llbracket \cup _\Sigma \{ \langle i, X\rangle \} \rrbracket _\mathfrak {A} \ne \emptyset$. (The union of the cells in a salvation chain does not represent the empty set.)

This semantics simply does not derive the semantic facts about a diagram from the semantic facts about the diagrams from which it is syntactically derived.

To see the difficulty that this can raise, we will show that this semantics cannot easily be made compositional, for reasons similar to those considered above. Suppose that Diagram *D* has been derived from diagram $D^\prime$, cell *X* and $i\in \mathbb {N}$ by applying **Salvation Rule**. What is wanted is some statement of the meaning of *D* along the lines of

- $$\llbracket D \rrbracket _\mathfrak {A} = f_{Salvation}(\llbracket D^\prime \rrbracket _\mathfrak {A},\llbracket X \rrbracket _\mathfrak {A},i)$$

However, the natural meaning to assign to $D^\prime$ (or, $\llbracket D^\prime \rrbracket _\mathfrak {A}$) is a truth-value. And, there is no obvious function from truth-values and the meanings of cells that yields the meaning of the diagram *D*. This is why in our cell-first semantics above we introduced that “existential assignment parameter” on analogy with introducing sequences into the semantics of first-order logic. Offering a compositional semantics, then, requires clarifying the representational commitments of the system.

We wish to be clear that our observation here is not meant as criticism of the standard semantics for Venn diagrams. These accounts were primarily developed to show that formal methods could be applied to Venn diagrams and that standard tools for analyzing proofs in formal languages could be applied to Venn diagrams. In this aim, they have been successful. In particular, Shin (1994) and Hammer and Danner (1996) demonstrate that Venn diagrams stand in formal entailment relations. Strictly speaking, this demonstration does not require considering whether or not the semantics is compositional.

However, because the focus has been on developing the logical features of Venn diagrams, the authors have not tried to develop a semantics for the interpretation of Venn diagrams that parallels the interpretation of language. As a result, their semantic proposals have many features that may appear to differ from the interpretation of linguistic representation. These differences may have made it appear that Venn diagrams and languages involve a different kind of representation. We believe that the discrepancy has tempted some to view the non-linguistic representation involved with Venn diagrams as exhibiting a kind of representation that is fundamentally different from linguistic representation—and that this difference can be generalized to other sorts of diagrammatic representation. We have shown, however, that the difference is incidental to the semantics. That is, there is a straightforward compositional semantics for Venn diagrams.^25^

Notice also that on the semantics we’ve given, the notions of truth and consequence are straightforward. First, as one might expect a diagram *D* is *true* in a model $\mathfrak {A}$ (i.e. $\mathfrak {A}$ is a model of *D*) if and only if $\llbracket D \rrbracket _\mathfrak {A}= 1$. We can write this as $\mathfrak {A} \vDash D$. Then a diagram *D* is a logical consequence of a set of diagrams Γ if and only if every model $\mathfrak {A}$ of Γ is also a model of *D*. That is: For a set of diagrams Γ and a diagram *D*, $\Gamma \vDash D$ iff for all $\mathfrak {A}$, if for all $D^* \in \Gamma$, $\mathfrak {A} \vDash D^*$, then $\mathfrak {A} \vDash D$.

### A note on Greenberg’s semantics for Euler diagrams

At this point, we should acknowledge that there are other semantics for diagrams on offer. Perhaps, these fare differently. One prominent example is Greenberg’s recent semantics for Euler diagrams (Greenberg, 2023, 593-6), the historical antecedents and close relatives of Venn diagrams (Euler, 1768). Greenberg’s semantics is very different from the sort normally offered for Venn (or Euler) diagrams in the literature. So, we will briefly look at it to see if it is compositional.

An *Euler diagram*, like a Venn diagram, is characterized by a number of overlapping regions. Euler diagrams differ from Venn diagrams primarily in that every displayed region is construed as representing a non-empty set, and the regions corresponding to empty sets are omitted. For example, Diagram 26 is a paradigmatic Euler diagram. It represents that there are *A*s, *B*s, and *C*s, and that the relations among these sets are as displayed: every *C* is an *A*, but not vice versa; some *B*s are *A*s, while others are not.

**Diagram 27:**
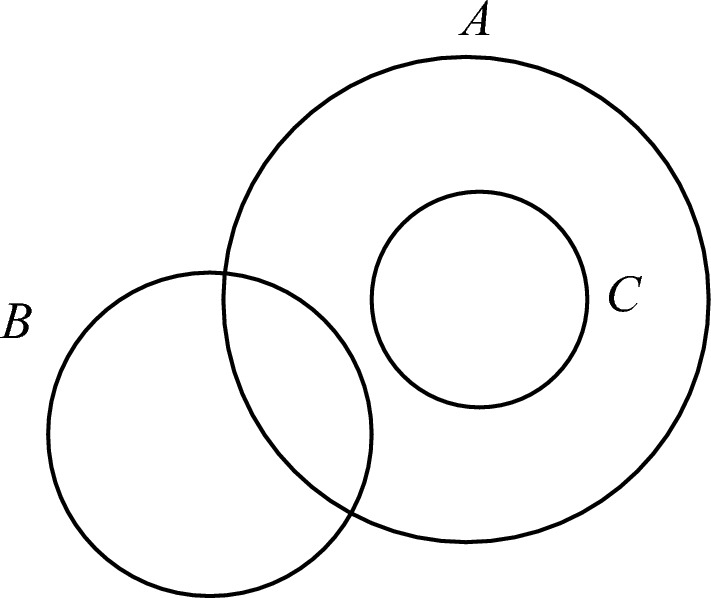
Euler diagram

We will briefly examine the approach to Euler diagrams offered by Greenberg (2023) as an alternative framework to the sort of approach to Venn diagrams developed both by standard accounts (Shin and Hammer and Danner ) and to our own framework. Greenberg’s approach differs from these more standard frameworks by cleaving the syntactic structure of diagrams from their semantic structure.

On his view, a diagram is not generated recursively from syntactic operations on regions. Instead, a diagram is stipulated to be a pair of (i) points and (ii) basic regions of points (possibly overlapping). The points and the basic regions are the *syntactic simples*. Semantically, points themselves are not assigned denotations, while each basic region *X* is directly assigned a denotation $\llbracket X \rrbracket$ (a subset of the domain). Thus the regions—and not the points—are the *semantic simples*. Notice that compositionality would rule out this approach by forcing us to align the syntactic and semantic simples. Although the points themselves are not given denotations, they do figure into the truth conditions: an Euler diagram *D* is true if there exists a function *f* mapping its points onto domain objects such that, for every point *p* in a region *X*, p∈X iff $f(p) \in \llbracket X \rrbracket$. This guarantees that all regions represent non-empty sets, though multiple points may map to the same object.

As we have mentioned, this approach makes points (i) syntactic simples of the diagrams and (ii) semantically active. However, it seems to us that the smallest components of Venn diagrams that are representationally relevant are regions, namely the cell regions. In the standard syntax and in our own, regions and whole diagrams are the inputs and outputs of the syntactic rules.

It may be true that regions are composed of points. But this is analogous to the fact that words and sentences are composed of letters. Letters have no syntactic or semantic significance for natural language beyond their role in identifying lexical items. Analogously, points have no syntactic or semantic significance in the semantics for Venn or Euler diagrams. Indeed, we have offered a syntax and semantics for Venn diagrams that makes no use of points.^26^ On our theory, all regions can be specified through set-theoretic operations on the basic regions. The denotations of these regions are similarly specified by set-theoretic operations on the denotations of the basic regions. The actual points involved in the diagram make no representational difference to the diagram. For example, the cardinality of the points in a region makes no representational difference.

This understanding of the role of points in diagrams seemingly reflects the attitude of Euler himself. On his view, spaces or regions correspond to the terms of a syllogism–one region might represent men, another mortals.

“As a general notion contains an infinite number of individual objects, we may consider it as a space in which they are all contained. Thus, for the notion of man we form a space [...] in which we conceive all men to be comprehended. For the notion of mortal we form another, [...] in which we conceive every thing mortal to be comprehended. And when I affirm, all men are mortal, it is the same thing with affirming, that the first figure is contained in the second” (Euler, 1768: 453)

Euler treats the entire region as the representational unit corresponding to a general concept (e.g., “man”, “mortal”), and the logical claim (“all men are mortal”) as a spatial containment relation between such regions. He does not treat points as representational, nor even as genuine constituents of the diagram. (This conception aligns with the classical view that space is not an aggregate of point-like elements but a continuous whole.)

Insofar as one assumes that there are points within a region, they are not part of the representational code. It’s wrong to think that in the region associated with the concept “man”, one point represents one man. The diagram is not a literal map of individuals. Instead, the space as a whole stands for the concept, and the relations between spaces stand for logical relations between concepts.^27^ Consequently, we regard centering points as syntactic or semantic components of the diagram as peculiar, if not misguided.^28^

## Conclusion

We began with the question of whether linguistic and visual representations are fundamentally different. It has been argued that visual representations lack syntactic structure or that, to the extent that visual representations exhibit syntactic structure, it is not the kind appropriate to compositional semantic theorizing of the sort applied to linguistic representation.

In this paper, we investigated Venn diagrams as a case study in how visual representations represent—or at least as a study of how a paradigmatic, yet well-behaved and constrained, visual system represents. We have developed two approaches to the recursive syntax of these diagrams: one region-first and the other cell-first. We then developed a rule-to-rule compositional semantics for each approach to syntax.

The two approaches to syntax give us different perspectives on the representational structure of Venn diagrams. On the region-first approach, the basic expressions are basic regions and other regions result from sequentially complementing, intersecting, or union-ing these regions. In this respect, the region-first approach might be seen as “top-down”. In contrast, the cell-first approach uses the basic regions to construct minimal regions or cells. The Venn diagram results from sequentially decorating these individual cells. In this respect, it might be seen as “bottom-up”. In contrast to the existing literature, we have clearly separated the two approaches.

Turning to the semantics, the extant semantic theories for Venn diagrams might have been potentially misleading, because they did not aim for compositionality. The meaning of a complex representation was not specified as a function of the meaning of the simpler representations from which it derived. Thus, the extant semantic theories do not look like the semantic theories on offer for linguistic representations. By developing compositional theories for Venn diagrams, we hope to have clarified this issue.

Of course, this does not rule out the possibility that there may be further fundamental differences between Venn diagrams and linguistic representations. Or, Venn diagrams might be unusually language-like visual representations. But, the two syntactic and semantics theories on offer may help to more precisely locate the similarities Venn diagrams share with linguistic representations and also their differences. There is room to explore how well the techniques developed here, including the two approaches to syntax, apply to other sorts of visual images, such as tactile Venn diagrams (Goldstein, 2011), pictures (e.g., Greenberg, 2021) or digital images (e.g., Isaac, 2017)—and, more generally, how they might apply to a diverse range of representational systems (cf., Schlenker, 2018).

## Appendix A

In order to walk through the full details of the cell-first syntax and semantics consider again Diagram 18 repeated below:

To derive this diagram start with the primary diagram $\texttt {form}(A_1,A_2,A_3)$ and then derive five cells. Call them *a*, *b*, *c*, *f*, and *g*:

- $$a = \texttt {cell}_{A_2,A_3}(A_1)$$
- $$b= \texttt {cell}_{A_3}(A_1,A_2)$$
- $$c= \texttt {cell}_{\emptyset }(A_1,A_2,A_3)$$
- $$f= \texttt {cell}_{A_1}(A_2,A_3)$$
- $$g= \texttt {cell}_{A_1,A_2}(A_3)$$

Given those cells of the diagram we then start decorating by destroying or saving cells. We destroy *c*, and then also destroy *b*:

- $$\texttt {destroy}(\texttt {form}(A_1,A_2,A_3), c)$$
- $$\texttt {destroy}(\texttt {destroy}(\texttt {form}(A_1,A_2,A_3), c),b)$$

We then save *a* by marking it with a ’2’, and then also save *f* by marking it with a ’1’:

- $$\texttt {save}(\texttt {destroy}(\texttt {destroy}(\texttt {form}(A_1,A_2,A_3), c),b),a,2)$$
- $$\texttt {save}(\texttt {save}(\texttt {destroy}(\texttt {destroy}(\texttt {form}(A_1,A_2,A_3), c),b),a,2),f,1)$$

Then we also save *g* by also marking it with a ’1’.

- $$\texttt {save}(\texttt {save}(\texttt {save}(\texttt {destroy}(\texttt {destroy}(\texttt {form}(A_1,A_2,A_3), c),b),a,2),f,1),g,1)$$

That’s the syntactic derivation of Diagram 18. Next we can compositionally derive its truth conditions as follows:

The final line follows given the semantics for each cell, i.e.:

$$\llbracket a\rrbracket ^{\sigma ^{\prime \prime }}_\mathfrak {A} = \llbracket \texttt {cell}_{ A_2,A_3} (A_1)\rrbracket ^{\sigma ^{\prime \prime }}_\mathfrak {A} = I(A_1) - \big (I(A_2)\cup I(A_3)\big )$$

$$\llbracket b\rrbracket ^{\sigma ^{\prime \prime \prime }}_\mathfrak {A} = \llbracket \texttt {cell}_{A_3} (A_1,A_2)\rrbracket ^{\sigma ^{\prime \prime \prime }}_\mathfrak {A} = \big (I(A_1)\cap I(A_2)\big ) - I(A_3)$$

$$\llbracket c\rrbracket ^{\sigma ^{\prime \prime \prime }}_\mathfrak {A} = \llbracket \texttt {cell}_{\emptyset } (A_1,A_2,A_3)\rrbracket ^{\sigma ^{\prime \prime \prime }}_\mathfrak {A} = \big (I(A_1)\cap I(A_2)\cap (A_3) \big )$$

$$\llbracket f\rrbracket ^{\sigma ^{\prime }}_\mathfrak {A} = \llbracket \texttt {cell}_{A_1} (A_2,A_3)\rrbracket ^{\sigma ^{\prime }}_\mathfrak {A} = \big (I(A_2)\cap I(A_3)\big ) - I(A_1)\big )$$

$$\llbracket g\rrbracket ^{\sigma }_\mathfrak {A} = \llbracket \texttt {cell}_{ A_1,A_2} (A_3)\rrbracket ^{\sigma }_\mathfrak {A} = I(A_3) - \big (I(A_1)\cup I(A_2)\big )$$

Thus, the diagram is true in a model $\mathfrak {A}=\langle U,I \rangle$ if and only if both $\big (I(A_3)-I(A_1)\big )$ and $\bigg (I(A_1) - \big (I(A_2)\cup I(A_3)\big )\bigg )$ are non-empty, and $\big (I(A_1)\cap I(A_2)\big )$ is empty.

## Footnotes

1 Camp (2007) argues that maps and diagrams and their constituents can be representations in a “language of thought”, but she insists that these representations are not “linguistic”. They are part of what she calls a *weak-LOT*: “a system of representational vehicles with some recurrent constituents that can be recombined according to some set of rules to produce representations of systematically related entire contents” (Camp, 2007: 152). See related discussion of maps (Blumson, 2012; Casati, 1999) and of images more generally (Blumson, 2010, 2014; Goodman, 1976; Greenberg, 2011, 2013; Kulvicki, 2006).

2 In this way, the approach in this paper makes a small contribution to the broader literature on representational formats—commonly discussed under the heading of a linguistic versus non-linguistic distinction, though the adequacy of that framing is itself contested, e.g., Shimojima (1999), Greenberg (2013), Blumson (2011, 2014), Rescorla (2009), Schlimm and Waszek (2020), Pietarinen (2017), Anger et al. (2022).

3 Here we draw on the interesting discussion of this history in Moktefi (2012) and especially Moktefi and Pietarinen (2015).

4 “We are quite able to meet [the demand to represent existential information], though, I must admit, with a certain loss of elegance and simplicity” (Venn, 1894: 130).

5 Notice that Diagram 8 represents what we could express with the *conjunction* of these formulae of first-order logic:

- $$\exists x((A_1(x) \wedge \lnot (A_2(x) \vee A_3(x))) \vee ((A_1(x) \wedge A_3(x)) \wedge \lnot A_2(x)))$$
- $$\exists x((A_3(x) \wedge \lnot (A_1(x) \vee A_2(x))) \vee ((A_1(x) \wedge A_3(x)) \wedge \lnot A_2(x)))$$
- $$\lnot \exists x(A_1(x) \wedge A_2(x) \wedge A_3(x))$$

6 Peirce’s convention is similar to Lewis Carroll’s (1897) “sitting-on-the-fence” convention, in which one puts an existential mark on the border between two cells.

7 For a range of implementations and contexts, see Hodges (1998), Pagin and Westerståhl (2010a, 2010b), Werning et al. (2012), Fodor and Lepore (2002), Pelletier (1994), and citations therein.

8 Stronger formulations, such as type-driven compositionality or composition via functional application, impose tighter constraints on how syntactic structure determines semantic interpretation. See Heim (1998) for an exposition in contemporary formal semantics.

9 Where η is a syntactic rule for deriving a complex representation from more basic representations, compositionality is formally stated as follows: If α = $\eta (\beta _1$,...,$\beta _n)$ and γ = $\eta (\delta _1$,...$,\delta _n)$, then if $\llbracket \beta _{i}\rrbracket$ = $\llbracket \delta _{i}\rrbracket$ (for all *i*), then $\llbracket \alpha \rrbracket$ = $\llbracket \gamma \rrbracket$. See standard definitions, and variants, in Pagin and Westerståhl (2010a).

10 As Partee puts it, “The relevant part-whole relation is given by the syntactic derivation in the syntax of the disambiguated language (‘rule-by-rule interpretation”). ‘Parts’ must be ‘immediate parts’ (derivationally)...” (Partee, 1984, 155).

11 A concrete illustration of how syntactic constituency constrains semantic composition is given by Partee (1973, 511–513), who discusses the NP ‘the boy who saw the girl’. The older “NP–S” analysis treated this phrase as formed from the NP ‘the boy’ plus the complement ‘who saw the girl’. On the more modern analysis, however, ‘boy’ first combines with the relative clause ‘who saw the girl’, and only then does the determiner ‘the’ apply. Partee argues that—given plausible assumptions about the relevant semantic values—only the latter analysis satisfies rule-to-rule compositionality, since the meanings of ‘the boy’ and ‘who saw the girl’ do not combine compositionally.

12 Weakened versions of compositionality (such as so-called “weak” or “global” compositionality which say that the meaning of a complex expression depends only on the meanings of its atomic expressions and their modes of combination) would allow for a semantic theory to depend on a non-constituent in this way. See Pagin and Westerståhl (2010a).

13 See Shin (1994: 57) and Hammer and Danner (1996: 466) where similar constraints are introduced, and compare with the formal definition of Venn diagrams in terms of *independent families* in Grünbaum (1975). To a certain extent, our way of proceeding departs from Shin and Hammer and Danner because their syntax specifies that from a diagram one may add another basic region under their “partial overlap” constraint. By forming the primary diagram all at once, we will specify the same set of diagrams while avoiding some complications.

14 In the syntax we have offered and in the standard syntactic theories, many Venn diagrams will have multiple derivations. For instance, a single region may be specified in terms of a relative complement or as a union of other regions, a destroyed region of a diagram may be specified by destroying the region all at once or by specifying and destroying each of its cells piecemeal, and the order of destructions and salvations does not matter for the output diagram. These failures of *unique readability* are familiar from semantics for formal and natural language. Frege’s semantics for his formal language, *Begriffsschrift*, is often cited as the motivating example of a compositional semantics. However, as this semantics is developed in *The Basic Laws of Arithmetic*, formulae of *Begriffsschrift* have many syntactic derivations (see Pickel, 2010, Pickel & Rabern, 2023, Trueman forthcoming). In the case of natural language, some standard frameworks that aspire to compositionality allow formulae to be multiply readable (Jacobson, 2014, chapter 5). Similar issues have also been discussed with diagrams such as Peirce’s α-graphs, where the same diagram can be constructed by distinct syntactic operations, e.g., by different orders of juxtaposition or iteration (see Schlimm & Waszek, 2020).

15 The assumption that a primary diagram is true on every model is not the same as the assumption that total destruction—i.e., the diagram where every region is marked as empty—is false on every model. In other words, we are not assuming that the models have to have a non-empty universe. An undecorated region is strictly *neutral* regarding whether anything in the domain corresponds to it (contra Euler diagrams, see §7.1). There might be something, but there also might not be. This is in line with the way Venn introduced the different sorts of decorations: “...we need three distinct sorts of indication: one for marking the compartments unconditionally destroyed; one for those unconditionally saved; and one for those as to the state of which we are left in doubt.” (Venn, 1894: 130)

16 A Python implementation of the syntax and compositional semantics developed here, including rule-based construction and model-theoretic evaluation of diagrams, is available in Rabern (2023).

17 See Shin (1994, 57, rules 4 and 5) and Hammer and Danner (1996, 466, rule 3)

18 Note, that the two extreme cases are possible where either sequence is empty. In which case $\texttt {cell}_{ Y_1,\ldots ,Y_k}\emptyset$ is the “outside” of the diagram, the region that overlaps with none of the basic regions of the diagram. And $\texttt {cell}_{\emptyset }(X_1,\ldots ,X_j)$ is the region that overlaps with all the basic regions of the diagram.

19 So, formally σ is an indexed family (or a function or sequence, depending on how one looks at it).

20 Logical consequence can similarly be defined as preservation of truth at a model. Thus, the definition of consequence will need to be modified on analogy with the modification of the definition of truth in a model. In particular, Diagram *D* is a consequence of a set of diagrams Γ if and only if for every model $\mathfrak {A}$, if for every $D^* \in \Gamma$, $\llbracket D^* \rrbracket ^\emptyset _\mathfrak {A}$ = 1, then $\llbracket D\rrbracket ^\emptyset _\mathfrak {A}$ = 1.

21 See, e.g., Janssen (1997), Rabern (2013), Yli-Vakkuri (2013), and Pickel and Rabern (2023).

22 It is worth noting that Peirce also suggested this sort of device with his “existential graphs”—here too argument positions are linked with wires. See Peirce (1903: 15–23).

23 See Fine (2003), Wehmeier (2018), and Pickel and Rabern (2016).

24 In particular, Shin’s representations make use of “infons” from situation semantics (see Devlin, 1991). However, the infons associated with a diagram simply summarize the information in our $\langle B, \Delta , \Sigma \rangle$ triples.

25 Our semantics has focused on compositionally deriving the set of models in which a diagram is true. One might worry that we haven’t actually derived truth “conditions” *per se*, since truth conditions are inherently modal (Yalcin, 2018: 339-340). But it is a trivial exercise to recast our semantics in, for example, a possible worlds framework where the values derived are set of worlds.

26 Our approach could be adapted to provide a compositional semantics for Euler diagrams by building in Euler’s presupposition that every displayed cell has a non-empty extension. Formally, this can be modeled by treating salvation as enforcing a global non-emptiness constraint on cells, rather than as an optional existential device. Hammer and Shin (1998) provide a semantics for a revised version of Euler’s original approach that drops this presupposition. In effect, Shin-Hammer-Eulerian diagrams are Venn diagrams without salvation, whereas original Euler diagrams impose the additional requirement that every undestroyed cell is occupied. We leave the Eulerian extension of our framework undeveloped here.

27 Thanks to an anonymous referee for pointing to this passage in Euler’s letter.

28 This relates to a point that Isaac (2017) makes about digital images. Isaac argues that sometimes rescaling a digital image does not make a difference to its representational content. We take no stand on Isaac’s principle as applied to, say, digital photographs. However, as applied to Venn diagrams (and Euler diagrams), we hold that the points making up the regions are not representationally significant and there is no reason to appeal to them in giving the semantics for the diagrams.
